# Supplementary material for: Teaching Practical Skills in Anesthesia, Intensive Care, Emergency and Pain Medicine—What Is Really Relevant for Medical Students? Results of a German National Survey of Nearly 3000 Anesthesiologists
Source: Healthcare (Basel). 2022 Nov 11;10(11):2260. doi: 10.3390/healthcare10112260 (PMC9690720; doi:10.3390/healthcare10112260)
Supplement: Supplementary file 1 [file healthcare-10-02260-s001.zip › Table S1.pdf]

Supplement Table S1

The following supplement tables show the rating of the individual learning objectives by the participating physicians depending on various factors (personal qualification, job, etc.).

Table of contents

1. Physicians in training 2

2. Specialists 6

3. Senior physicians 10

4. Chief physicians 14

5. Retired physicians 18

6. Hospital for low level of care 22

7. Hospital for advanced level of care 26

8. Hospital with maximum level of care 30

9. University hospital 34

10. Hospital with special supply 38

11. Rehabilitation hospital 42

12. Outpatient anesthesia practice 46

13. Additional certification in emergency medicine 50

14. Additional certification in intensive care medicine 54

15. Additional certification in pain medicine 58

16. Additional certification in palliative care 62

1. Physicians in training

The following table shows the rating of the individual learning objectives by the participating physicians in training.

Table 1.1: Overview of all items asked in the individual categories in the field of anesthesiology and the average rating of the participants from 1 ("not relevant at all") to 6 ("very relevant") as median and IQR and mean value and standard deviation

item	category	competence	n	median	IQR	mean	SD
At the end of undergraduate training, the student, as an active member of the professional team, can safely carry out clinical-practical skills adequately and independently under supervision, in a manner that is respectful of the patient. The student can…							
	**premedication visit**						
**1**	taking patient history relevant to anesthesia		339	3.00	2.00	3.12	1.29
**2**	performing an anesthesia focused physical examination (auscultation of heart/lung, status of teeth, predictors of a difficult airway. …)		330	4.00	2.00	3.77	1.39
**3**	performing a 12-channel-ecg and interpretation of the result		336	5.00	2.00	4.89	1.27
**4**	conducting an informed consent discussion with an ASAI/ASAII patient undergoing a low to medium risk operation and documenting it in a legally correct manner		336	2.00	2.00	2.38	1.27
	**preparation of general anesthesia**						
**5**	performing a quick check of the anesthesia working place according to the recommendations of DGAI		307	1.00	1.00	1.77	1.02
**6**	increasing patient safety by completing a standardized preoperative check list (e.g. WHO check list)		305	4.00	2.00	3.82	1.64
**7**	establishing intraoperative monitoring (ecg, non-invasive blood pressure monitoring, temperature, relaxometry, pulse oximetry/oxygen saturation)		306	5.00	2.00	4.65	1.35
**8**	setting up an iv-drip for infusion		304	5.00	2.00	5.21	1.25
**9**	preparing drugs for intravenous application		302	6.00	1.00	4.97	1.38

**10**	establishing a peripheral iv catheter	304	6.00	2.00	5.36	1.08
**11**	establishing a central iv catheter	305	6.00	1.00	2.07	1.05
**12**	establishing an arterial catheter	301	2.00	2.00	2.16	1.12
**13**	applying drugs intravenously, intramuscularly, subcutaneously	302	2.00	2.00	4.79	1.47
	**general anesthesia**					
**14**	being capable of a sufficient preoxygenation	296	3.00	3.00	3.48	1.57
**15**	being able to induce a general anesthesia using hypnotics. opioids and muscle relaxants with adequate dosing	296	2.00	2.00	2.03	1.13
**16**	being able to open the upper respiratory tract by using the Esmarch manoeuvre	296	5.00	3.00	4.33	1.70
**17**	being capable of ventilating a patient with a face mask (may be using a supraglottic airway tube)	294	4.00	3.00	4.07	1.73
**18**	knowing how to correctly insert a laryngeal mask airway and checking for its correct positioning	295	3.00	2.00	2.82	1.49
**19**	knowing how to correctly insert a laryngeal tube and checking for its correct positioning	296	2.00	3.00	2.67	1.54
**20**	intubating a patient and checking for the correct endotracheal positioning	296	2.00	2.00	2.05	1.17
**21**	performing the initial steps of an emergency algorithm when encountering an unexpected difficult airway	295	2.00	2.00	2.48	1.54
**22**	setting up an adequate mechanical ventilation according to the patient and the operation	294	2.00	2.00	1.95	1.07
	**regional anesthesia and pain therapy**					
**23**	taking patient history focused on pain symptoms	284	4.00	2.00	4.07	1.43
**24**	setting up a therapy plan according to the WHO analgesic ladder	283	4.00	2.00	3.93	1.47
**25**	being accustomed to the usage of patient-controlled anesthesia devices (PCA)	283	2.00	1.00	1.86	0.96

**26**	performing spinal anesthesia	283	1.00	0.00	1.30	0.66
**27**	performing epidural anesthesia	281	1.00	0.00	1.19	0.49
**28**	performing combined spinal/epidural anesthesia (CSE)	278	1.00	0.00	1.12	0.40
	accomplishing a peripheral nerve block by…					
**29**	an interscalene approach to the brachial plexus	284	1.00	0.00	1.10	0.34
**30**	a supraclavicular approach to the brachial plexus	284	1.00	0.00	1.08	0.31
**31**	an axillary approach to the brachial plexus	284	1.00	0.00	1.12	0.42
**32**	blocking the femoral nerve	283	1.00	0.00	1.14	0.43
**33**	blocking the sciatic nerve with a proximal approach	283	1.00	0.00	1.08	0.33
**34**	blocking the sciatic nerve with distal approach	283	1.00	0.00	1.10	0.37

Table 1.2: Overview of all items asked in the individual categories in the field of intensive care medicine and emergency medicine and the average rating of the participants from 1 ("not relevant at all") to 6 ("very relevant") as median and IQR and mean value and standard deviation

	**category**	**competence**	**n**	**median**	**IQR**	**mean**	**SD**
At the end of undergraduate training, the student, as an active member of the professional team, can safely carry out clinical-practical skills adequately and independently under supervision, in a manner that is respectful of the patient. The student can…							
	**intensive care unit**						
**35**	performing a clinically focused physical exam		277	6.00	2.00	5.02	1.24
**36**	transferring information regarding a patient among healthcare professionals utilizing a defined technique (for example SBAR)		277	5.00	2.00	4.58	1.44
**37**	managing an analgosedation for an intervention		275	2.00	2.00	2.34	1.17
**38**	assessing the depth of sedation of a patient using an established scoring system		276	2.00	2.00	2.59	1.44
**39**	demonstrating ultrasound examination utilizing the eFAST principle		278	3.00	2.00	2.98	1.62
**40**	inserting a gastric tube		277	3.00	2.00	3.51	1.49
**41**	inserting a urinary catheter		277	3.00	2.00	3.38	1.59

**42**	performing a bronchoscopy on an intubated patient	277	1.00	1.00	1.42	0.79
**43**	evacuating air by puncturing of a tension pneumothorax	277	2.00	3.00	2.76	1.66
**44**	puncturing and/or drainage of intrapleural fluids	278	2.00	2.00	2.05	1.15
**45**	performing an minithoracotomy and placing a chest tube	277	1.00	1.00	1.76	1.18
**46**	obtaining blood samples for microbiological examination	278	5.00	2.00	4.91	1.37
**47**	performing a blood transfusion according to current guidelines	278	5.00	2.00	4.75	1.58
**48**	calling a patient's death	277	5.00	2.00	4.78	1.54
**49**	inspecting a corpse externally	277	5.00	3.00	4.36	1.68
**50**	completing a death certificate and correctly differentiating the cause of death	277	4.00	4.00	3.91	1.79
**51**	communication adequately with patients and/or relatives in crisis situations	278	4.00	3.00	3.98	1.72
	**emergency medicine**					
**52**	calculating the Glasgow Coma Scale	279	6.00	2.00	4.95	1.32
**53**	evaluating a patient using the ABCDE system	277	6.00	2.00	4.96	1.35
**54**	performing a rapid sequence induction and intubation	277	1.00	1.00	1.80	1.05
**55**	establishing an intraosseous needle	278	2.00	3.00	2.67	1.52
**56**	bringing a patient into lateral recumbent position	279	6.00	0.00	5.58	0.97
**57**	stabilizing the cervical vertebrae using a stifneck	278	5.00	2.00	4.67	1.53
**58**	immobilizing a patient using a vacuum mattress or spineboard	279	3.00	3.00	3.50	1.64
**59**	placing a pelvic binder	279	3.00	3.00	3.36	1.64
**60**	placing a tourniquet	278	4.00	3.00	3.66	1.74
**61**	diagnosing a cardiac arrest	277	6.00	0.00	5.47	1.19
**62**	utilizing the basic life support algorithms according to current guidelines and performing effective chest compressions	279	6.00	0.00	5.54	1.03
	utilizing the advanced life support algorithms according to current guidelines and…					
**63**	correctly analysing the different rhythms in cardiac arrest	279	6.00	2.00	4.87	1.45
**64**	correctly perform defibrillation/cardioversion	277	6.00	2.00	4.86	1.49
**65**	correctly administer drugs	277	5.00	2.00	4.76	1.54

2. Specialists

The following table shows the rating of the individual learning objectives by the participating specialists.

Table 2.1: Overview of all items asked in the individual categories in the field of anesthesiology and the average rating of the participants from 1 ("not relevant at all") to 6 ("very relevant") as median and IQR and mean value and standard deviation

item	category	competence	n	median	IQR	mean	SD
At the end of undergraduate training, the student, as an active member of the professional team, can safely carry out clinical-practical skills adequately and independently under supervision, in a manner that is respectful of the patient. The student can…							
	**premedication visit**						
**1**	taking patient history relevant to anesthesia		714	3.00	2.00	3.37	1.40
**2**	performing an anesthesia focused physical examination (auscultation of heart/lung, status of teeth, predictors of a difficult airway. …)		700	4.00	2.00	3.72	1.40
**3**	performing a 12-channel-ecg and interpretation of the result		712	5.00	2.00	4.58	1.38
**4**	conducting an informed consent discussion with an ASAI/ASAII patient undergoing a low to medium risk operation and documenting it in a legally correct manner		711	2.00	2.00	2.51	1.32
	**preparation of general anesthesia**						
**5**	performing a quick check of the anesthesia working place according to the recommendations of DGAI		668	2.00	1.00	1.99	1.31
**6**	increasing patient safety by completing a standardized preoperative check list (e.g. WHO check list)		663	4.00	2.00	4.03	1.67
**7**	establishing intraoperative monitoring (ecg, non-invasive blood pressure monitoring, temperature, relaxometry, pulse oximetry/oxygen saturation)		662	5.00	2.00	4.41	1.45
**8**	setting up an iv-drip for infusion		662	6.00	1.00	5.13	1.27
**9**	preparing drugs for intravenous application		662	6.00	2.00	4.93	1.39

**10**	establishing a peripheral iv catheter	662	6.00	1.00	5.18	1.20
**11**	establishing a central iv catheter	665	2.00	2.00	1.88	1.07
**12**	establishing an arterial catheter	656	2.00	2.00	1.82	1.04
**13**	applying drugs intravenously. intramuscularly. subcutaneously	665	5.00	2.00	4.49	1.51
	**general anesthesia**					
**14**	being capable of a sufficient preoxygenation	639	4.00	3.00	3.77	1.62
**15**	being able to induce a general anesthesia using hypnotics. opioids and muscle relaxants with adequate dosing	634	2.00	2.00	1.97	1.12
**16**	being able to open the upper respiratory tract by using the Esmarch manoeuvre	640	5.00	2.00	4.67	1.56
**17**	being capable of ventilating a patient with a face mask (may be using a supraglottic airway tube)	634	4.50	3.00	4.22	1.67
**18**	knowing how to correctly insert a laryngeal mask airway and checking for its correct positioning	640	3.00	2.00	2.91	1.52
**19**	knowing how to correctly insert a laryngeal tube and checking for its correct positioning	635	3.00	3.00	2.83	1.59
**20**	intubating a patient and checking for the correct endotracheal positioning	636	2.00	2.00	2.13	1.22
**21**	performing the initial steps of an emergency algorithm when encountering an unexpected difficult airway	637	2.00	3.00	2.50	1.51
**22**	setting up an adequate mechanical ventilation according to the patient and the operation	635	2.00	2.00	1.93	1.15
	**regional anesthesia and pain therapy**					
**23**	taking patient history focused on pain symptoms	626	4.00	2.00	3.78	1.54
**24**	setting up a therapy plan according to the WHO analgesic ladder	618	4.00	3.00	3.60	1.51
**25**	being accustomed to the usage of patient-controlled anesthesia devices (PCA)	617	2.00	2.00	1.98	1.18

**26**	performing spinal anesthesia	620	1.00	0.00	1.31	0.70
**27**	performing epidural anesthesia	621	1.00	0.00	1.21	0.61
**28**	performing combined spinal/epidural anesthesia (CSE)	619	1.00	0.00	1.16	0.53
	accomplishing a peripheral nerve block by…					
**29**	an interscalene approach to the brachial plexus	624	1.00	0.00	1.16	0.51
**30**	a supraclavicular approach to the brachial plexus	623	1.00	0.00	1.15	0.49
**31**	an axillary approach to the brachial plexus	623	1.00	0.00	1.21	0.60
**32**	blocking the femoral nerve	622	1.00	0.00	1.23	0.63
**33**	blocking the sciatic nerve with a proximal approach	621	1.00	0.00	1.18	0.56
**34**	blocking the sciatic nerve with distal approach	617	1.00	0.00	1.19	0.56

Table 2.3: Overview of all items asked in the individual categories in the field of intensive care medicine and emergency medicine and the average rating of the participants from 1 ("not relevant at all") to 6 ("very relevant") as median and IQR and mean value and standard deviation

	**category**	**competence**	**n**	**median**	**IQR**	**mean**	**SD**
At the end of undergraduate training, the student, as an active member of the professional team, can safely carry out clinical-practical skills adequately and independently under supervision, in a manner that is respectful of the patient. The student can…							
	**intensive care unit**						
**35**	performing a clinically focused physical exam		609	5.00	2.00	4.74	1.44
**36**	transferring information regarding a patient among healthcare professionals utilizing a defined technique (for example SBAR)		604	4.00	3.00	4.19	1.60
**37**	managing an analgosedation for an intervention		607	2.00	2.00	2.17	1.19
**38**	assessing the depth of sedation of a patient using an established scoring system		609	2.00	2.00	2.74	1.47
**39**	demonstrating ultrasound examination utilizing the eFAST principle		608	2.00	2.00	2.42	1.45
**40**	inserting a gastric tube		609	3.00	3.00	3.42	1.62
**41**	inserting a urinary catheter		610	3.00	3.00	3.40	1.69

**42**	performing a bronchoscopy on an intubated patient	610	1.00	1.00	1.43	0.82
**43**	evacuating air by puncturing of a tension pneumothorax	609	2.00	3.00	2.73	1.70
**44**	puncturing and/or drainage of intrapleural fluids	611	2.00	2.00	2.00	1.20
**45**	performing an minithoracotomy and placing a chest tube	605	1.00	1.00	1.71	1.10
**46**	obtaining blood samples for microbiological examination	607	5.00	3.00	4.44	1.59
**47**	performing a blood transfusion according to current guidelines	610	5.00	3.00	4.30	1.75
**48**	calling a patient's death	610	5.00	3.00	4.53	1.67
**49**	inspecting a corpse externally	610	4.00	4.00	3.97	1.84
**50**	completing a death certificate and correctly differentiating the cause of death	608	4.00	4.00	3.73	1.88
**51**	communication adequately with patients and/or relatives in crisis situations	610	4.00	3.25	3.79	1.74
	**emergency medicine**					
**52**	calculating the Glasgow Coma Scale	597	6.00	2.00	4.87	1.43
**53**	evaluating a patient using the ABCDE system	588	5.00	2.00	4.79	1.47
**54**	performing a rapid sequence induction and intubation	595	1.00	1.00	1.87	1.18
**55**	establishing an intraosseous needle	591	2.00	3.00	2.76	1.64
**56**	bringing a patient into lateral recumbent position	597	6.00	0.00	5.48	1.08
**57**	stabilizing the cervical vertebrae using a stifneck	595	5.00	2.00	4.72	1.53
**58**	immobilizing a patient using a vacuum mattress or spineboard	597	4.00	3.00	3.96	1.68
**59**	placing a pelvic binder	594	3.00	3.00	3.49	1.70
**60**	placing a tourniquet	593	4.00	3.00	3.95	1.71
**61**	diagnosing a cardiac arrest	594	6.00	0.00	5.50	1.07
**62**	utilizing the basic life support algorithms according to current guidelines and performing effective chest compressions	595	6.00	1.00	5.44	1.15
	utilizing the advanced life support algorithms according to current guidelines and…					
**63**	correctly analysing the different rhythms in cardiac arrest	595	5.00	2.00	4.68	1.49
**64**	correctly perform defibrillation/cardioversion	594	5.00	2.00	4.69	1.53
**65**	correctly administer drugs	594	5.00	2.00	4.66	1.53

3. Senior physicians

The following table shows the rating of the individual learning objectives by the participating senior physicians.

Table 3.1: Overview of all items asked in the individual categories in the field of anesthesiology and the average rating of the participants from 1 ("not relevant at all") to 6 ("very relevant") as median and IQR and mean value and standard deviation

item	category	competence	n	mean	SD	median	IQR
At the end of undergraduate training, the student, as an active member of the professional team, can safely carry out clinical-practical skills adequately and independently under supervision, in a manner that is respectful of the patient. The student can…							
	**premedication visit**						
**1**	taking patient history relevant to anesthesia		823	3.00	2.00	3.31	1.41
**2**	performing an anesthesia focused physical examination (auscultation of heart/lung, status of teeth, predictors of a difficult airway, …)		803	4.00	2.00	3.78	1.41
**3**	performing a 12-channel-ecg and interpretation of the result		816	5.00	2.00	4.66	1.37
**4**	conducting an informed consent discussion with an ASAI/ASAII patient undergoing a low to medium risk operation and documenting it in a legally correct manner		814	2.00	2.00	2.55	1.37
	**preparation of general anesthesia**						
**5**	performing a quick check of the anesthesia working place according to the recommendations of DGAI		780	1.00	2	2.00	1.36
**6**	increasing patient safety by completing a standardized preoperative check list (e.g. WHO check list)		767	4.00	3	4.16	1.66
**7**	establishing intraoperative monitoring (ecg, non-invasive blood pressure monitoring, temperature, relaxometry, pulse oximetry/oxygen saturation)		773	4.00	3	4.23	1.49
**8**	setting up an iv-drip for infusion		773	6.00	2	5.06	1.35
**9**	preparing drugs for intravenous application		767	6.00	2	4.97	1.38

**10**	establishing a peripheral iv catheter	766	6.00	2	5.12	1.23
**11**	establishing a central iv catheter	771	2.00	1	1.85	1.05
**12**	establishing an arterial catheter	761	2.00	1	1.82	1.04
**13**	applying drugs intravenously, intramuscularly, subcutaneously	771	5.00	3	4.55	1.52
	**general anesthesia**					
**14**	being capable of a sufficient preoxygenation	748	4.00	3.00	3.55	1.67
**15**	being able to induce a general anesthesia using hypnotics. opioids and muscle relaxants with adequate dosing	741	2.00	2.00	1.99	1.17
**16**	being able to open the upper respiratory tract by using the Esmarch manoeuvre	746	5.00	3.00	4.53	1.61
**17**	being capable of ventilating a patient with a face mask (may be using a supraglottic airway tube)	747	4.00	3.00	4.07	1.69
**18**	knowing how to correctly insert a laryngeal mask airway and checking for its correct positioning	745	2.00	3.00	2.74	1.53
**19**	knowing how to correctly insert a laryngeal tube and checking for its correct positioning	744	2.00	3.00	2.63	1.56
**20**	intubating a patient and checking for the correct endotracheal positioning	743	2.00	2.00	2.06	1.19
**21**	performing the initial steps of an emergency algorithm when encountering an unexpected difficult airway	747	2.00	2.00	2.38	1.45
**22**	setting up an adequate mechanical ventilation according to the patient and the operation	743	2.00	1.00	1.88	1.08
	**regional anesthesia and pain therapy**					
**23**	taking patient history focused on pain symptoms	735	4.00	2.00	3.70	1.48
**24**	setting up a therapy plan according to the WHO analgesic ladder	729	4.00	3.00	3.61	1.50
**25**	being accustomed to the usage of patient-controlled anesthesia devices (PCA)	731	2.00	2.00	1.95	1.14

**26**	performing spinal anesthesia	734	1.00	0.00	1.34	0.77
**27**	performing epidural anesthesia	733	1.00	0.00	1.21	0.60
**28**	performing combined spinal/epidural anesthesia (CSE)	735	1.00	0.00	1.13	0.51
	accomplishing a peripheral nerve block by…					
**29**	an interscalene approach to the brachial plexus	728	1.00	0.00	1.12	0.47
**30**	a supraclavicular approach to the brachial plexus	737	1.00	0.00	1.11	0.44
**31**	an axillary approach to the brachial plexus	733	1.00	0.00	1.15	0.52
**32**	blocking the femoral nerve	737	1.00	0.00	1.17	0.55
**33**	blocking the sciatic nerve with a proximal approach	735	1.00	0.00	1.12	0.44
**34**	blocking the sciatic nerve with distal approach	735	1.00	0.00	1.13	0.48

Table 3.4: Overview of all items asked in the individual categories in the field of intensive care medicine and emergency medicine and the average rating of the participants from 1 ("not relevant at all") to 6 ("very relevant") as median and IQR and mean value and standard deviation

	**category**	**competence**	**n**	**median**	**IQR**	**mean**	**SD**
At the end of undergraduate training, the student, as an active member of the professional team, can safely carry out clinical-practical skills adequately and independently under supervision, in a manner that is respectful of the patient. The student can…							
	**intensive care unit**						
**35**	performing a clinically focused physical exam		716	5.00	2.00	4.72	1.37
**36**	transferring information regarding a patient among healthcare professionals utilizing a defined technique (for example SBAR)		713	4.00	3.00	4.16	1.59
**37**	managing an analgosedation for an intervention		710	2.00	2.00	2.11	1.20
**38**	assessing the depth of sedation of a patient using an established scoring system		715	2.00	3.00	2.71	1.50
**39**	demonstrating ultrasound examination utilizing the eFAST principle		716	2.00	2.00	2.48	1.47
**40**	inserting a gastric tube		716	3.00	3.00	3.57	1.60
**41**	inserting a urinary catheter		712	3.00	3.00	3.50	1.71

**42**	performing a bronchoscopy on an intubated patient	714	1.00	0.00	1.37	0.77
**43**	evacuating air by puncturing of a tension pneumothorax	712	2.00	3.00	2.83	1.73
**44**	puncturing and/or drainage of intrapleural fluids	715	2.00	2.00	1.99	1.18
**45**	performing an minithoracotomy and placing a chest tube	710	1.00	1.00	1.65	1.06
**46**	obtaining blood samples for microbiological examination	713	5.00	3.00	4.54	1.55
**47**	performing a blood transfusion according to current guidelines	713	5.00	3.00	4.27	1.79
**48**	calling a patient's death	714	5.00	3.00	4.47	1.77
**49**	inspecting a corpse externally	714	4.00	4.00	3.88	1.88
**50**	completing a death certificate and correctly differentiating the cause of death	712	4.00	4.00	3.70	1.89
**51**	communication adequately with patients and/or relatives in crisis situations	714	4.00	3.00	3.66	1.67
	**emergency medicine**					
**52**	calculating the Glasgow Coma Scale	708	6.00	2.00	4.94	1.37
**53**	evaluating a patient using the ABCDE system	691	5.00	2.00	4.76	1.48
**54**	performing a rapid sequence induction and intubation	706	1.00	1.00	1.74	1.07
**55**	establishing an intraosseous needle	705	2.00	3.00	2.56	1.59
**56**	bringing a patient into lateral recumbent position	705	6.00	0.00	5.43	1.18
**57**	stabilizing the cervical vertebrae using a stifneck	705	5.00	2.00	4.72	1.52
**58**	immobilizing a patient using a vacuum mattress or spineboard	705	4.00	3.00	3.94	1.71
**59**	placing a pelvic binder	703	3.00	3.00	3.40	1.71
**60**	placing a tourniquet	702	4.00	4.00	3.87	1.74
**61**	diagnosing a cardiac arrest	706	6.00	0.00	5.42	1.19
**62**	utilizing the basic life support algorithms according to current guidelines and performing effective chest compressions	706	6.00	1.00	5.37	1.20
	utilizing the advanced life support algorithms according to current guidelines and…					
**63**	correctly analysing the different rhythms in cardiac arrest	706	5.00	3.00	4.54	1.53
**64**	correctly perform defibrillation/cardioversion	706	5.00	3.00	4.58	1.58
**65**	correctly administer drugs	705	5.00	3.00	4.49	1.60

4. Chief physicians

The following table shows the rating of the individual learning objectives by the participating chief physicians/heads of department.

Table 4.1: Overview of all items asked in the individual categories in the field of anesthesiology and the average rating of the participants from 1 ("not relevant at all") to 6 ("very relevant") as median and IQR and mean value and standard deviation

item	category	competence	n	median	IQR	mean	SD
At the end of undergraduate training, the student, as an active member of the professional team, can safely carry out clinical-practical skills adequately and independently under supervision, in a manner that is respectful of the patient. The student can…							
	**premedication visit**						
**1**	taking patient history relevant to anesthesia		274	3.00	2.00	3.15	1.47
**2**	performing an anesthesia focused physical examination (auscultation of heart/lung, status of teeth, predictors of a difficult airway, …)		268	3.00	3.00	3.59	1.52
**3**	performing a 12-channel-ecg and interpretation of the result		271	5.00	2.00	4.59	1.39
**4**	conducting an informed consent discussion with an ASAI/ASAII patient undergoing a low to medium risk operation and documenting it in a legally correct manner		275	2.00	2.00	2.48	1.40
	**preparation of general anesthesia**						
**5**	performing a quick check of the anesthesia working place according to the recommendations of DGAI		264	1.00	1.00	1.92	1.43
**6**	increasing patient safety by completing a standardized preoperative check list (e.g. WHO check list)		262	4.00	3.00	4.13	1.64
**7**	establishing intraoperative monitoring (ecg, non-invasive blood pressure monitoring, temperature, relaxometry, pulse oximetry/oxygen saturation)		260	4.00	2.00	3.90	1.56
**8**	setting up an iv-drip for infusion		260	5.00	2.00	4.77	1.45
**9**	preparing drugs for intravenous application		262	5.00	2.00	4.73	1.51

**10**	establishing a peripheral iv catheter	261	5.00	2.00	4.78	1.40
**11**	establishing a central iv catheter	260	1.00	1.00	1.73	1.05
**12**	establishing an arterial catheter	260	1.00	1.00	1.70	1.10
**13**	applying drugs intravenously. intramuscularly. subcutaneously	263	5.00	3.00	4.47	1.54
	**general anesthesia**					
**14**	being capable of a sufficient preoxygenation	252	3.00	3.00	3.42	1.65
**15**	being able to induce a general anesthesia using hypnotics. opioids and muscle relaxants with adequate dosing	252	1.00	1.00	1.83	1.21
**16**	being able to open the upper respiratory tract by using the Esmarch manoeuvre	256	5.00	3.00	4.31	1.64
**17**	being capable of ventilating a patient with a face mask (may be using a supraglottic airway tube)	255	4.00	4.00	3.81	1.70
**18**	knowing how to correctly insert a laryngeal mask airway and checking for its correct positioning	255	2.00	3.00	2.60	1.54
**19**	knowing how to correctly insert a laryngeal tube and checking for its correct positioning	253	2.00	3.00	2.45	1.57
**20**	intubating a patient and checking for the correct endotracheal positioning	255	2.00	2.00	2.04	1.34
**21**	performing the initial steps of an emergency algorithm when encountering an unexpected difficult airway	255	2.00	2.00	2.27	1.53
**22**	setting up an adequate mechanical ventilation according to the patient and the operation	254	1.00	1.00	1.74	1.13
	**regional anesthesia and pain therapy**					
**23**	taking patient history focused on pain symptoms	253	3.00	3.00	3.56	1.46
**24**	setting up a therapy plan according to the WHO analgesic ladder	249	4.00	3.00	3.60	1.46
**25**	being accustomed to the usage of patient-controlled anesthesia devices (PCA)	250	2.00	2.00	1.97	1.25

**26**	performing spinal anesthesia	250	1.00	0.00	1.32	0.76
**27**	performing epidural anesthesia	249	1.00	0.00	1.21	0.68
**28**	performing combined spinal/epidural anesthesia (CSE)	247	1.00	0.00	1.16	0.64
	accomplishing a peripheral nerve block by…					
**29**	an interscalene approach to the brachial plexus	250	1.00	0.00	1.18	0.64
**30**	a supraclavicular approach to the brachial plexus	251	1.00	0.00	1.16	0.56
**31**	an axillary approach to the brachial plexus	249	1.00	0.00	1.24	0.75
**32**	blocking the femoral nerve	248	1.00	0.00	1.27	0.78
**33**	blocking the sciatic nerve with a proximal approach	249	1.00	0.00	1.16	0.56
**34**	blocking the sciatic nerve with distal approach	245	1.00	0.00	1.19	0.61

Table 4.5: Overview of all items asked in the individual categories in the field of intensive care medicine and emergency medicine and the average rating of the participants from 1 ("not relevant at all") to 6 ("very relevant") as median and IQR and mean value and standard deviation

	**category**	**competence**	**n**	**median**	**IQR**	**mean**	**SD**
At the end of undergraduate training, the student, as an active member of the professional team, can safely carry out clinical-practical skills adequately and independently under supervision, in a manner that is respectful of the patient. The student can…							
	**intensive care unit**						
**35**	performing a clinically focused physical exam		248	5.00	3.00	4.48	1.55
**36**	transferring information regarding a patient among healthcare professionals utilizing a defined technique (for example SBAR)		247	4.00	3.00	3.71	1.71
**37**	managing an analgosedation for an intervention		249	2.00	2.00	2.07	1.25
**38**	assessing the depth of sedation of a patient using an established scoring system		245	2.00	2.00	2.61	1.48
**39**	demonstrating ultrasound examination utilizing the eFAST principle		250	2.00	2.00	2.42	1.50
**40**	inserting a gastric tube		250	3.00	3.00	3.28	1.69
**41**	inserting a urinary catheter		249	3.00	3.00	3.49	1.73

**42**	performing a bronchoscopy on an intubated patient	249	1.00	0.00	1.34	0.82
**43**	evacuating air by puncturing of a tension pneumothorax	250	2.00	3.00	2.78	1.75
**44**	puncturing and/or drainage of intrapleural fluids	249	2.00	2.00	2.02	1.27
**45**	performing an minithoracotomy and placing a chest tube	248	1.00	1.00	1.66	1.15
**46**	obtaining blood samples for microbiological examination	249	5.00	3.00	4.24	1.64
**47**	performing a blood transfusion according to current guidelines	250	5.00	3.00	4.13	1.83
**48**	calling a patient's death	249	5.00	3.00	4.30	1.81
**49**	inspecting a corpse externally	249	4.00	4.00	3.80	1.95
**50**	completing a death certificate and correctly differentiating the cause of death	248	4.00	4.00	3.65	1.96
**51**	communication adequately with patients and/or relatives in crisis situations	249	3.00	3.00	3.47	1.80
	**emergency medicine**					
**52**	calculating the Glasgow Coma Scale	246	5.00	3.00	4.67	1.53
**53**	evaluating a patient using the ABCDE system	244	5.00	3.00	4.47	1.61
**54**	performing a rapid sequence induction and intubation	246	1.00	1.00	1.72	1.25
**55**	establishing an intraosseous needle	243	2.00	2.00	2.40	1.51
**56**	bringing a patient into lateral recumbent position	244	6.00	1.00	5.26	1.32
**57**	stabilizing the cervical vertebrae using a stifneck	245	5.00	2.00	4.60	1.62
**58**	immobilizing a patient using a vacuum mattress or spineboard	245	4.00	3.00	4.03	1.72
**59**	placing a pelvic binder	246	3.00	2.00	3.14	1.70
**60**	placing a tourniquet	244	4.00	3.00	3.79	1.68
**61**	diagnosing a cardiac arrest	246	6.00	1.00	5.33	1.23
**62**	utilizing the basic life support algorithms according to current guidelines and performing effective chest compressions	243	6.00	1.00	5.28	1.33
	utilizing the advanced life support algorithms according to current guidelines and…					
**63**	correctly analysing the different rhythms in cardiac arrest	244	5.00	3.00	4.48	1.55
**64**	correctly perform defibrillation/cardioversion	245	5.00	3.00	4.44	1.69
**65**	correctly administer drugs	246	5.00	3.00	4.38	1.64

5. Retired physicians

The following table shows the rating of the individual learning objectives by the participating retired physicians.

Table 5.1: Overview of all items asked in the individual categories in the field of anesthesiology and the average rating of the participants from 1 ("not relevant at all") to 6 ("very relevant") as median and IQR and mean value and standard deviation

item	category	competence	n	median	IQR	mean	SD
At the end of undergraduate training, the student, as an active member of the professional team, can safely carry out clinical-practical skills adequately and independently under supervision, in a manner that is respectful of the patient. The student can…							
	**premedication visit**						
**1**	taking patient history relevant to anesthesia		144	3.00	2.00	3.28	1.42
**2**	performing an anesthesia focused physical examination (auscultation of heart/lung, status of teeth, predictors of a difficult airway, …)		134	4.00	2.00	3.73	1.48
**3**	performing a 12-channel-ecg and interpretation of the result		140	4.00	2.00	4.00	1.49
**4**	conducting an informed consent discussion with an ASAI/ASAII patient undergoing a low to medium risk operation and documenting it in a legally correct manner		142	3.00	1.25	2.73	1.40
	**preparation of general anesthesia**						
**5**	performing a quick check of the anesthesia working place according to the recommendations of DGAI		132	1.50	2.00	1.94	1.20
**6**	increasing patient safety by completing a standardized preoperative check list (e.g. WHO check list)		130	4.00	3.00	3.75	1.67
**7**	establishing intraoperative monitoring (ecg, non-invasive blood pressure monitoring, temperature, relaxometry, pulse oximetry/oxygen saturation)		133	4.00	2.00	3.85	1.55
**8**	setting up an iv-drip for infusion		133	5.00	2.00	4.83	1.36
**9**	preparing drugs for intravenous application		130	5.00	3.00	4.65	1.46

**10**	establishing a peripheral iv catheter	131	5.00	2.00	4.66	1.44
**11**	establishing a central iv catheter	130	1.00	1.00	1.87	1.20
**12**	establishing an arterial catheter	127	1.00	1.00	1.74	1.22
**13**	applying drugs intravenously, intramuscularly, subcutaneously	131	5.00	3.00	4.44	1.56
	**general anesthesia**					
**14**	being capable of a sufficient preoxygenation	127	4.00	3.00	3.72	1.66
**15**	being able to induce a general anesthesia using hypnotics. opioids and muscle relaxants with adequate dosing	130	1.00	1.00	1.84	1.22
**16**	being able to open the upper respiratory tract by using the Esmarch manoeuvre	128	5.00	3.00	4.52	1.61
**17**	being capable of ventilating a patient with a face mask (may be using a supraglottic airway tube)	129	4.00	3.00	3.70	1.73
**18**	knowing how to correctly insert a laryngeal mask airway and checking for its correct positioning	128	3.00	2.00	2.89	1.57
**19**	knowing how to correctly insert a laryngeal tube and checking for its correct positioning	125	2.00	3.00	2.71	1.59
**20**	intubating a patient and checking for the correct endotracheal positioning	126	2.00	2.00	2.21	1.36
**21**	performing the initial steps of an emergency algorithm when encountering an unexpected difficult airway	128	2.00	3.00	2.50	1.58
**22**	setting up an adequate mechanical ventilation according to the patient and the operation	126	1.00	2.00	1.94	1.26
	**regional anesthesia and pain therapy**					
**23**	taking patient history focused on pain symptoms	125	4.00	2.00	3.79	1.43
**24**	setting up a therapy plan according to the WHO analgesic ladder	123	3.00	2.00	3.41	1.49
**25**	being accustomed to the usage of patient-controlled anesthesia devices (PCA)	125	2.00	2.00	2.14	1.24
**26**	performing spinal anesthesia	124	1.00	0.00	1.42	0.88
**27**	performing epidural anesthesia	123	1.00	0.00	1.33	0.81

**28**	performing combined spinal/epidural anesthesia (CSE)	124	1.00	0.00	1.25	0.72
	accomplishing a peripheral nerve block by…					
**29**	an interscalene approach to the brachial plexus	124	1.00	0.00	1.27	0.71
**30**	a supraclavicular approach to the brachial plexus	122	1.00	0.00	1.26	0.73
**31**	an axillary approach to the brachial plexus	121	1.00	0.00	1.43	0.97
**32**	blocking the femoral nerve	121	1.00	0.00	1.40	0.88
**33**	blocking the sciatic nerve with a proximal approach	120	1.00	0.00	1.26	0.74
**34**	blocking the sciatic nerve with distal approach	118	1.00	0.00	1.26	0.75

Table 5.6: Overview of all items asked in the individual categories in the field of intensive care medicine and emergency medicine and the average rating of the participants from 1 ("not relevant at all") to 6 ("very relevant") as median and IQR and mean value and standard deviation

	**category**	**competence**	**n**	**median**	**IQR**	**mean**	**SD**
At the end of undergraduate training, the student, as an active member of the professional team, can safely carry out clinical-practical skills adequately and independently under supervision, in a manner that is respectful of the patient. The student can…							
	**intensive care unit**						
**35**	performing a clinically focused physical exam		121	5.00	3.00	4.34	1.48
**36**	transferring information regarding a patient among healthcare professionals utilizing a defined technique (for example SBAR)		120	4.00	3.00	3.54	1.61
**37**	managing an analgosedation for an intervention		121	2.00	2.00	2.13	1.32
**38**	assessing the depth of sedation of a patient using an established scoring system		122	2.00	3.00	2.66	1.51
**39**	demonstrating ultrasound examination utilizing the eFAST principle		122	2.00	2.00	2.27	1.44
**40**	inserting a gastric tube		122	4.00	4.00	3.87	1.65
**41**	inserting a urinary catheter		121	4.00	4.00	3.88	1.72
**42**	performing a bronchoscopy on an intubated patient		122	1.00	1.00	1.50	1.05
**43**	evacuating air by puncturing of a tension pneumothorax		122	3.00	4.00	3.04	1.84

**44**	puncturing and/or drainage of intrapleural fluids	122	2.00	2.00	2.34	1.39
**45**	performing an minithoracotomy and placing a chest tube	122	1.00	2.00	1.87	1.32
**46**	obtaining blood samples for microbiological examination	121	5.00	3.00	4.30	1.61
**47**	performing a blood transfusion according to current guidelines	121	4.00	4.00	3.97	1.86
**48**	calling a patient's death	121	5.00	3.00	4.30	1.78
**49**	inspecting a corpse externally	122	4.00	4.00	3.84	1.93
**50**	completing a death certificate and correctly differentiating the cause of death	122	4.00	4.00	3.78	1.91
**51**	communication adequately with patients and/or relatives in crisis situations	121	4.00	4.00	3.68	1.87
	**emergency medicine**					
**52**	calculating the Glasgow Coma Scale	117	4.00	3.00	4.28	1.63
**53**	evaluating a patient using the ABCDE system	116	4.00	3.00	4.18	1.59
**54**	performing a rapid sequence induction and intubation	117	1.00	2.00	1.79	1.21
**55**	establishing an intraosseous needle	117	2.00	3.00	2.61	1.70
**56**	bringing a patient into lateral recumbent position	118	6.00	0.00	5.51	1.08
**57**	stabilizing the cervical vertebrae using a stifneck	118	6.00	2.00	5.07	1.38
**58**	immobilizing a patient using a vacuum mattress or spineboard	118	5.00	3.00	4.42	1.67
**59**	placing a pelvic binder	117	3.00	2.00	3.56	1.78
**60**	placing a tourniquet	117	5.00	3.00	4.38	1.71
**61**	diagnosing a cardiac arrest	117	6.00	0.00	5.43	1.15
**62**	utilizing the basic life support algorithms according to current guidelines and performing effective chest compressions	118	6.00	1.00	5.21	1.26
	utilizing the advanced life support algorithms according to current guidelines and…					
**63**	correctly analysing the different rhythms in cardiac arrest	117	5.00	3.00	4.36	1.61
**64**	correctly perform defibrillation/cardioversion	116	5.00	3.00	4.37	1.67
**65**	correctly administer drugs	117	5.00	3.00	4.35	1.66

6. Hospital for low level of care

The following table shows the rating of the individual learning objectives by the participating physicians working in regional hospitals with low level of care.

Table 6.1: Overview of all items asked in the individual categories in the field of anesthesiology and the average rating of the participants from 1 ("not relevant at all") to 6 ("very relevant") as median and IQR and mean value and standard deviation

item	category	competence	n	median	IQR	mea n	SD
At the end of undergraduate training, the student, as an active member of the professional team, can safely carry out clinical-practical skills adequately and independently under supervision, in a manner that is respectful of the patient. The student can…							
	**premedication visit**						
**1**	taking patient history relevant to anesthesia		593	3.00	2.00	3.19	1.38
**2**	performing an anesthesia focused physical examination (auscultation of heart/lung, status of teeth, predictors of a difficult airway, …)		576	4.00	2.75	3.63	1.43
**3**	performing a 12-channel-ecg and interpretation of the result		587	5.00	2.00	4.52	1.35
**4**	conducting an informed consent discussion with an ASAI/ASAII patient undergoing a low to medium risk operation and documenting it in a legally correct manner		586	2.00	2.00	2.50	1.34
	**preparation of general anesthesia**						
**5**	performing a quick check of the anesthesia working place according to the recommendations of DGAI		557	1.00	1.00	1.89	1.29
**6**	increasing patient safety by completing a standardized preoperative check list (e.g. WHO check list)		554	4.00	3.00	4.01	1.70
**7**	establishing intraoperative monitoring (ecg, non-invasive blood pressure monitoring, temperature, relaxometry, pulse oximetry/oxygen saturation)		555	4.00	3.00	4.23	1.51
**8**	setting up an iv-drip for infusion		558	6.00	2.00	5.09	1.30
**9**	preparing drugs for intravenous application		554	6.00	2.00	4.91	1.39
**10**	establishing a peripheral iv catheter		554	6.00	2.00	5.07	1.28

**11**	establishing a central iv catheter	554	2.00	1.00	1.86	1.05
**12**	establishing an arterial catheter	551	1.00	1.00	1.83	1.08
**13**	applying drugs intravenously, intramuscularly, subcutaneously	556	5.00	3.00	4.48	1.55
	**general anesthesia**					
**14**	being capable of a sufficient preoxygenation	540	4.00	3.00	3.63	1.65
**15**	being able to induce a general anesthesia using hypnotics. opioids and muscle relaxants with adequate dosing	540	2.00	3.00	1.86	1.05
**16**	being able to open the upper respiratory tract by using the Esmarch manoeuvre	540	5.00	3.00	4.51	1.59
**17**	being capable of ventilating a patient with a face mask (may be using a supraglottic airway tube)	541	4.00	3.00	4.02	1.65
**18**	knowing how to correctly insert a laryngeal mask airway and checking for its correct positioning	542	2.50	3.00	2.78	1.58
**19**	knowing how to correctly insert a laryngeal tube and checking for its correct positioning	539	3.00	3.00	2.83	1.60
**20**	intubating a patient and checking for the correct endotracheal positioning	540	2.00	2.00	2.11	1.29
**21**	performing the initial steps of an emergency algorithm when encountering an unexpected difficult airway	542	2.00	2.00	2.38	1.48
**22**	setting up an adequate mechanical ventilation according to the patient and the operation	539	1.00	1.00	1.78	1.04
	**regional anesthesia and pain therapy**					
**23**	taking patient history focused on pain symptoms	532	4.00	2.00	3.76	1.44
**24**	setting up a therapy plan according to the WHO analgesic ladder	524	4.00	3.00	3.59	1.44
**25**	being accustomed to the usage of patient-controlled anesthesia devices (PCA)	531	2.00	2.00	1.95	1.16
**26**	performing spinal anesthesia	527	1.00	0.00	1.28	0.68
**27**	performing epidural anesthesia	530	1.00	0.00	1.17	0.54

**28**	performing combined spinal/epidural anesthesia (CSE)	528	1.00	0.00	1.11	0.42
	accomplishing a peripheral nerve block by…					
**29**	an interscalene approach to the brachial plexus	528	1.00	0.00	1.11	0.42
**30**	a supraclavicular approach to the brachial plexus	527	1.00	0.00	1.10	0.40
**31**	an axillary approach to the brachial plexus	528	1.00	0.00	1.14	0.47
**32**	blocking the femoral nerve	527	1.00	0.00	1.18	0.54
**33**	blocking the sciatic nerve with a proximal approach	526	1.00	0.00	1.11	0.41
**34**	blocking the sciatic nerve with distal approach	521	1.00	0.00	1.13	0.46

Table 6.7: Overview of all items asked in the individual categories in the field of intensive care medicine and emergency medicine and the average rating of the participants from 1 ("not relevant at all") to 6 ("very relevant") as median and IQR and mean value and standard deviation

	**category**	**competence**	**n**	**mean**	**IQR**	**mean**	**SD**
At the end of undergraduate training, the student, as an active member of the professional team, can safely carry out clinical-practical skills adequately and independently under supervision, in a manner that is respectful of the patient. The student can…							
	**intensive care unit**						
**35**	performing a clinically focused physical exam		515	5.00	2.00	4.68	1.41
**36**	transferring information regarding a patient among healthcare professionals utilizing a defined technique (for example SBAR)		513	4.00	3.00	4.08	1.63
**37**	managing an analgosedation for an intervention		513	2.00	2.00	2.11	1.19
**38**	assessing the depth of sedation of a patient using an established scoring system		513	2.00	3.00	2.63	1.46
**39**	demonstrating ultrasound examination utilizing the eFAST principle		513	2.00	2.00	2.47	1.47
**40**	inserting a gastric tube		515	3.00	3.00	3.53	1.59
**41**	inserting a urinary catheter		516	3.00	3.00	3.51	1.67
**42**	performing a bronchoscopy on an intubated patient		513	1.00	0.00	1.34	0.71
**43**	evacuating air by puncturing of a tension pneumothorax		513	2.00	3.00	2.80	1.68

**44**	puncturing and/or drainage of intrapleural fluids	516	2.00	2.00	2.03	1.19
**45**	performing an minithoracotomy and placing a chest tube	511	1.00	1.00	1.70	1.11
**46**	obtaining blood samples for microbiological examination	512	5.00	2.00	4.60	1.52
**47**	performing a blood transfusion according to current guidelines	512	5.00	3.00	4.27	1.80
**48**	calling a patient's death	515	5.00	3.00	4.57	1.69
**49**	inspecting a corpse externally	513	4.00	3.00	4.09	1.82
**50**	completing a death certificate and correctly differentiating the cause of death	512	4.00	4.00	3.85	1.87
**51**	communication adequately with patients and/or relatives in crisis situations	513	4.00	3.00	3.71	1.71
	**emergency medicine**					
**52**	calculating the Glasgow Coma Scale	506	5.00	2.00	4.83	1.40
**53**	evaluating a patient using the ABCDE system	500	5.00	2.00	4.69	1.50
**54**	performing a rapid sequence induction and intubation	505	1.00	1.00	1.75	1.13
**55**	establishing an intraosseous needle	502	2.00	3.00	2.59	1.59
**56**	bringing a patient into lateral recumbent position	507	6.00	1.00	5.45	1.15
**57**	stabilizing the cervical vertebrae using a stifneck	505	5.00	2.00	4.73	1.55
**58**	immobilizing a patient using a vacuum mattress or spineboard	507	4.00	3.00	4.02	1.72
**59**	placing a pelvic binder	503	3.00	3.00	3.39	1.71
**60**	placing a tourniquet	504	4.00	4.00	3.90	1.71
**61**	diagnosing a cardiac arrest	507	6.00	0.00	5.47	1.10
**62**	utilizing the basic life support algorithms according to current guidelines and performing effective chest compressions	508	6.00	1.00	5.38	1.19
	utilizing the advanced life support algorithms according to current guidelines and…					
**63**	correctly analysing the different rhythms in cardiac arrest	505	5.00	2.00	4.57	1.50
**64**	correctly perform defibrillation/cardioversion	507	5.00	2.00	4.62	1.53
**65**	correctly administer drugs	506	5.00	2.24	4.55	1.54

7. Hospital for advanced level of care

The following table shows the rating of the individual learning objectives by the participating physicians working in overregional hospitals with advanced level of care.

Table 7.1: Overview of all items asked in the individual categories in the field of anesthesiology and the average rating of the participants from 1 ("not relevant at all") to 6 ("very relevant") as median and IQR and mean value and standard deviation

item	category	competence	n	median	IQR	mean	SD
At the end of undergraduate training, the student, as an active member of the professional team, can safely carry out clinical-practical skills adequately and independently under supervision, in a manner that is respectful of the patient. The student can…							
	**premedication visit**						
**1**	taking patient history relevant to anesthesia		489	3.00	2.00	3.15	1.32
**2**	performing an anesthesia focused physical examination (auscultation of heart/lung, status of teeth, predictors of a difficult airway, …)		471	4.00	2.00	3.65	1.41
**3**	performing a 12-channel-ecg and interpretation of the result		483	5.00	2.00	4.52	1.40
**4**	conducting an informed consent discussion with an ASAI/ASAII patient undergoing a low to medium risk operation and documenting it in a legally correct manner		488	2.00	2.00	2.47	1.34
	**preparation of general anesthesia**						
**5**	performing a quick check of the anesthesia working place according to the recommendations of DGAI		468	1.00	1.00	1.84	1.27
**6**	increasing patient safety by completing a standardized preoperative check list (e.g. WHO check list)		459	4.00	3.00	4.05	1.64
**7**	establishing intraoperative monitoring (ecg, non-invasive blood pressure monitoring, temperature, relaxometry, pulse oximetry/oxygen saturation)		463	4.00	3.00	4.12	1.55
**8**	setting up an iv-drip for infusion		459	6.00	2.00	4.93	1.39
**9**	preparing drugs for intravenous application		459	5.00	2.00	4.82	1.44
**10**	establishing a peripheral iv catheter		459	6.00	2.00	4.98	1.28

**11**	establishing a central iv catheter	460	2.00	1.00	1.84	1.06
**12**	establishing an arterial catheter	453	1.00	1.00	1.83	1.09
**13**	applying drugs intravenously, intramuscularly, subcutaneously	462	5.00	3.00	4.41	1.54
	**general anesthesia**					
**14**	being capable of a sufficient preoxygenation	442	3.00	3.00	3.45	1.68
**15**	being able to induce a general anesthesia using hypnotics. opioids and muscle relaxants with adequate dosing	439	1.00	1.00	1.87	1.16
**16**	being able to open the upper respiratory tract by using the Esmarch manoeuvre	444	5.00	3.00	4.29	1.66
**17**	being capable of ventilating a patient with a face mask (may be using a supraglottic airway tube)	440	4.00	4.00	3.82	1.75
**18**	knowing how to correctly insert a laryngeal mask airway and checking for its correct positioning	441	2.00	3.00	2.61	1.48
**19**	knowing how to correctly insert a laryngeal tube and checking for its correct positioning	437	2.00	2.00	2.38	1.47
**20**	intubating a patient and checking for the correct endotracheal positioning	439	2.00	2.00	1.99	1.21
**21**	performing the initial steps of an emergency algorithm when encountering an unexpected difficult airway	441	2.00	2.00	2.27	1.49
**22**	setting up an adequate mechanical ventilation according to the patient and the operation	436	1.00	1.00	1.79	1.11
	**regional anesthesia and pain therapy**					
**23**	taking patient history focused on pain symptoms	433	4.00	3.00	3.62	1.52
**24**	setting up a therapy plan according to the WHO analgesic ladder	430	4.00	3.00	3.59	1.54
**25**	being accustomed to the usage of patient-controlled anesthesia devices (PCA)	430	1.00	2.00	1.90	1.16
**26**	performing spinal anesthesia	430	1.00	0.00	1.34	0.76
**27**	performing epidural anesthesia	427	1.00	0.00	1.20	0.61

**28**	performing combined spinal/epidural anesthesia (CSE)	427	1.00	0.00	1.13	0.52
	accomplishing a peripheral nerve block by…					
**29**	an interscalene approach to the brachial plexus	433	1.00	0.00	1.15	0.54
**30**	a supraclavicular approach to the brachial plexus	432	1.00	0.00	1.15	0.54
**31**	an axillary approach to the brachial plexus	431	1.00	0.00	1.21	0.66
**32**	blocking the femoral nerve	428	1.00	0.00	1.20	0.66
**33**	blocking the sciatic nerve with a proximal approach	430	1.00	0.00	1.16	0.57
**34**	blocking the sciatic nerve with distal approach	426	1.00	0.00	1.16	0.57

Table 7.8: Overview of all items asked in the individual categories in the field of intensive care medicine and emergency medicine and the average rating of the participants from 1 ("not relevant at all") to 6 ("very relevant") as median and IQR and mean value and standard deviation

	**category**	**competence**	**n**	**median**	**IQR**	**mean**	**SD**
At the end of undergraduate training, the student, as an active member of the professional team, can safely carry out clinical-practical skills adequately and independently under supervision, in a manner that is respectful of the patient. The student can…							
	**intensive care unit**						
**35**	performing a clinically focused physical exam		424	5.00	2.00	4.65	1.45
**36**	transferring information regarding a patient among healthcare professionals utilizing a defined technique (for example SBAR)		423	4.00	3.00	3.98	1.67
**37**	managing an analgosedation for an intervention		424	2.00	2.00	2.15	1.23
**38**	assessing the depth of sedation of a patient using an established scoring system		423	2.00	2.00	2.64	1.46
**39**	demonstrating ultrasound examination utilizing the eFAST principle		424	2.00	2.00	2.47	1.52
**40**	inserting a gastric tube		424	3.00	3.00	3.42	1.63
**41**	inserting a urinary catheter		421	3.00	3.00	3.41	1.71
**42**	performing a bronchoscopy on an intubated patient		423	1.00	1.00	1.39	0.82
**43**	evacuating air by puncturing of a tension pneumothorax		421	2.00	3.00	2.68	1.70

**44**	puncturing and/or drainage of intrapleural fluids	423	2.00	2.00	1.98	1.22
**45**	performing an minithoracotomy and placing a chest tube	419	1.00	1.00	1.62	1.08
**46**	obtaining blood samples for microbiological examination	421	5.00	3.00	4.39	1.57
**47**	performing a blood transfusion according to current guidelines	423	5.00	3.00	4.27	1.74
**48**	calling a patient's death	423	5.00	3.00	4.48	1.73
**49**	inspecting a corpse externally	423	4.00	4.00	3.87	1.91
**50**	completing a death certificate and correctly differentiating the cause of death	421	4.00	4.00	3.67	1.92
**51**	communication adequately with patients and/or relatives in crisis situations	424	4.00	3.00	3.63	1.69
	**emergency medicine**					
**52**	calculating the Glasgow Coma Scale	417	5.00	2.00	4.78	1.42
**53**	evaluating a patient using the ABCDE system	414	5.00	3.00	4.63	1.52
**54**	performing a rapid sequence induction and intubation	417	1.00	1.00	1.63	1.01
**55**	establishing an intraosseous needle	416	2.00	3.00	2.48	1.58
**56**	bringing a patient into lateral recumbent position	415	6.00	1.00	5.38	1.21
**57**	stabilizing the cervical vertebrae using a stifneck	415	5.00	3.00	4.59	1.56
**58**	immobilizing a patient using a vacuum mattress or spineboard	414	4.00	4.00	3.80	1.73
**59**	placing a pelvic binder	416	3.00	2.00	3.18	1.67
**60**	placing a tourniquet	414	4.00	3.00	3.73	1.74
**61**	diagnosing a cardiac arrest	416	6.00	1.00	5.36	1.21
**62**	utilizing the basic life support algorithms according to current guidelines and performing effective chest compressions	413	6.00	1.00	5.32	1.26
	utilizing the advanced life support algorithms according to current guidelines and…					
**63**	correctly analysing the different rhythms in cardiac arrest	415	5.00	3.00	4.46	1.53
**64**	correctly perform defibrillation/cardioversion	415	5.00	3.00	4.46	1.63
**65**	correctly administer drugs	416	5.00	3.00	4.34	1.62

8. Hospital with maximum level of care

The following table shows the rating of the individual learning objectives by the participating physicians working in overregional hospitals with maximum level of care.

Table 8.1: Overview of all items asked in the individual categories in the field of anesthesiology and the average rating of the participants from 1 ("not relevant at all") to 6 ("very relevant") as median and IQR and mean value and standard deviation

item	category	competence	n	median	IQR	mean	SD
At the end of undergraduate training, the student, as an active member of the professional team, can safely carry out clinical-practical skills adequately and independently under supervision, in a manner that is respectful of the patient. The student can…							
	**premedication visit**						
**1**	taking patient history relevant to anesthesia		359	3.00	2.00	3.15	1.41
**2**	performing an anesthesia focused physical examination (auscultation of heart/lung, status of teeth, predictors of a difficult airway, …)		345	4.00	2.00	3.72	1.45
**3**	performing a 12-channel-ecg and interpretation of the result		349	5.00	2.00	4.63	1.40
**4**	conducting an informed consent discussion with an ASAI/ASAII patient undergoing a low to medium risk operation and documenting it in a legally correct manner		350	2.00	2.00	2.39	1.30
	**preparation of general anesthesia**						
**5**	performing a quick check of the anesthesia working place according to the recommendations of DGAI		334	1.00	1.00	1.85	1.18
**6**	increasing patient safety by completing a standardized preoperative check list (e.g. WHO check list)		328	4.00	3.00	4.07	1.73
**7**	establishing intraoperative monitoring (ecg, non-invasive blood pressure monitoring, temperature, relaxometry, pulse oximetry/oxygen saturation)		329	5.00	3.00	4.36	1.46
**8**	setting up an iv-drip for infusion		328	6.00	2.00	5.05	1.36
**9**	preparing drugs for intravenous application		327	6.00	2.00	4.88	1.48
**10**	establishing a peripheral iv catheter		327	6.00	2.00	5.17	1.24

**11**	establishing a central iv catheter	330	2.00	2.00	1.90	1.04
**12**	establishing an arterial catheter	323	2.00	2.00	1.92	1.07
**13**	applying drugs intravenously, intramuscularly, subcutaneously	327	5.00	2.00	4.63	1.47
	**general anesthesia**					
**14**	being capable of a sufficient preoxygenation	320	3.00	3.00	3.45	1.69
**15**	being able to induce a general anesthesia using hypnotics. opioids and muscle relaxants with adequate dosing	317	2.00	2.00	1.97	1.11
**16**	being able to open the upper respiratory tract by using the Esmarch manoeuvre	321	5.00	3.00	4.41	1.72
**17**	being capable of ventilating a patient with a face mask (may be using a supraglottic airway tube)	318	4.00	3.25	4.00	1.75
**18**	knowing how to correctly insert a laryngeal mask airway and checking for its correct positioning	321	3.00	3.00	2.73	1.48
**19**	knowing how to correctly insert a laryngeal tube and checking for its correct positioning	319	2.00	3.00	2.62	1.56
**20**	intubating a patient and checking for the correct endotracheal positioning	317	2.00	2.00	2.03	1.14
**21**	performing the initial steps of an emergency algorithm when encountering an unexpected difficult airway	317	2.00	2.00	2.37	1.45
**22**	setting up an adequate mechanical ventilation according to the patient and the operation	317	2.00	2.00	1.94	1.11
	**regional anesthesia and pain therapy**					
**23**	taking patient history focused on pain symptoms	314	4.00	2.00	3.77	1.50
**24**	setting up a therapy plan according to the WHO analgesic ladder	308	4.00	3.00	3.62	1.49
**25**	being accustomed to the usage of patient-controlled anesthesia devices (PCA)	308	2.00	1.00	1.82	1.02
**26**	performing spinal anesthesia	313	1.00	0.00	1.27	0.65
**27**	performing epidural anesthesia	312	1.00	0.00	1.18	0.56

**28**	performing combined spinal/epidural anesthesia (CSE)	307	1.00	0.00	1.13	0.46
	accomplishing a peripheral nerve block by…					
**29**	an interscalene approach to the brachial plexus	313	1.00	0.00	1.12	0.48
**30**	a supraclavicular approach to the brachial plexus	310	1.00	0.00	1.11	0.48
**31**	an axillary approach to the brachial plexus	312	1.00	0.00	1.18	0.60
**32**	blocking the femoral nerve	312	1.00	0.00	1.16	0.55
**33**	blocking the sciatic nerve with a proximal approach	310	1.00	0.00	1.10	0.46
**34**	blocking the sciatic nerve with distal approach	310	1.00	0.00	1.13	0.50

Table 8.9: Overview of all items asked in the individual categories in the field of intensive care medicine and emergency medicine and the average rating of the participants from 1 ("not relevant at all") to 6 ("very relevant") as median and IQR and mean value and standard deviation

	**category**	**competence**	**n**	**median**	**IQR**	**mean**	**SD**
At the end of undergraduate training, the student, as an active member of the professional team, can safely carry out clinical-practical skills adequately and independently under supervision, in a manner that is respectful of the patient. The student can…							
	**intensive care unit**						
**35**	performing a clinically focused physical exam		311	5.00	2.00	4.84	1.30
**36**	transferring information regarding a patient among healthcare professionals utilizing a defined technique (for example SBAR)		305	4.00	3.00	4.17	1.54
**37**	managing an analgosedation for an intervention		309	2.00	2.00	2.12	1.15
**38**	assessing the depth of sedation of a patient using an established scoring system		310	2.00	2.00	2.62	1.49
**39**	demonstrating ultrasound examination utilizing the eFAST principle		311	2.00	3.00	2.63	1.57
**40**	inserting a gastric tube		309	3.00	3.00	3.51	1.56
**41**	inserting a urinary catheter		309	3.00	3.00	3.36	1.69
**42**	performing a bronchoscopy on an intubated patient		312	1.00	1.00	1.40	0.80
**43**	evacuating air by puncturing of a tension pneumothorax		311	2.00	3.00	2.77	1.75

**44**	puncturing and/or drainage of intrapleural fluids	311	2.00	2.00	2.00	1.23
**45**	performing an minithoracotomy and placing a chest tube	310	1.00	1.00	1.69	1.13
**46**	obtaining blood samples for microbiological examination	312	5.00	3.00	4.65	1.51
**47**	performing a blood transfusion according to current guidelines	310	5.00	3.00	4.44	1.71
**48**	calling a patient's death	310	5.00	3.00	4.60	1.70
**49**	inspecting a corpse externally	311	4.00	3.00	4.09	1.79
**50**	completing a death certificate and correctly differentiating the cause of death	311	4.00	4.00	3.85	1.83
**51**	communication adequately with patients and/or relatives in crisis situations	312	4.00	3.00	3.66	1.76
	**emergency medicine**					
**52**	calculating the Glasgow Coma Scale	309	6.00	2.00	4.92	1.47
**53**	evaluating a patient using the ABCDE system	303	6.00	2.00	4.82	1.50
**54**	performing a rapid sequence induction and intubation	305	1.00	1.00	1.76	1.11
**55**	establishing an intraosseous needle	307	2.00	3.00	2.61	1.62
**56**	bringing a patient into lateral recumbent position	308	6.00	0.00	5.49	1.12
**57**	stabilizing the cervical vertebrae using a stifneck	308	5.00	2.00	4.74	1.52
**58**	immobilizing a patient using a vacuum mattress or spineboard	309	4.00	3.00	3.93	1.72
**59**	placing a pelvic binder	307	3.00	3.00	3.50	1.74
**60**	placing a tourniquet	306	4.00	4.00	3.90	1.77
**61**	diagnosing a cardiac arrest	306	6.00	0.00	5.40	1.21
**62**	utilizing the basic life support algorithms according to current guidelines and performing effective chest compressions	309	6.00	1.00	5.38	1.21
	utilizing the advanced life support algorithms according to current guidelines and…					
**63**	correctly analysing the different rhythms in cardiac arrest	309	5.00	3.00	4.65	1.54
**64**	correctly perform defibrillation/cardioversion	305	5.00	3.00	4.62	1.64
**65**	correctly administer drugs	304	5.00	3.00	4.63	1.60

9. University hospital

The following table shows the rating of the individual learning objectives by the participating physicians working in university hospitals.

Table 9.1: Overview of all items asked in the individual categories in the field of anesthesiology and the average rating of the participants from 1 ("not relevant at all") to 6 ("very relevant") as median and IQR and mean value and standard deviation

item	category	competence	n	median	IQR	mean	SD
At the end of undergraduate training, the student, as an active member of the professional team, can safely carry out clinical-practical skills adequately and independently under supervision, in a manner that is respectful of the patient. The student can…							
	**premedication visit**						
**1**	taking patient history relevant to anesthesia		489	3.00	2.00	3.53	1.41
**2**	performing an anesthesia focused physical examination (auscultation of heart/lung, status of teeth, predictors of a difficult airway, …)		483	4.00	2.00	3.93	1.35
**3**	performing a 12-channel-ecg and interpretation of the result		489	6.00	2.00	5.03	1.25
**4**	conducting an informed consent discussion with an ASAI/ASAII patient undergoing a low to medium risk operation and documenting it in a legally correct manner		489	2.00	1.00	2.60	1.32
	**preparation of general anesthesia**						
**5**	performing a quick check of the anesthesia working place according to the recommendations of DGAI		455	2.00	2.00	2.11	1.35
**6**	increasing patient safety by completing a standardized preoperative check list (e.g. WHO check list)		453	4.00	3.00	4.17	1.56
**7**	establishing intraoperative monitoring (ecg, non-invasive blood pressure monitoring, temperature, relaxometry, pulse oximetry/oxygen saturation)		453	5.00	2.00	4.53	1.38
**8**	setting up an iv-drip for infusion		452	6.00	2.00	5.13	1.28
**9**	preparing drugs for intravenous application		449	6.00	2.00	4.97	1.38
**10**	establishing a peripheral iv catheter		447	6.00	1.00	5.27	1.13

**11**	establishing a central iv catheter	451	2.00	2.00	1.97	1.09
**12**	establishing an arterial catheter	445	2.00	2.00	1.97	1.10
**13**	applying drugs intravenously, intramuscularly, subcutaneously	450	5.00	2.00	4.68	1.44
	**general anesthesia**					
**14**	being capable of a sufficient preoxygenation	437	4.00	3.00	3.66	1.58
**15**	being able to induce a general anesthesia using hypnotics. opioids and muscle relaxants with adequate dosing	437	2.00	2.00	2.13	1.22
**16**	being able to open the upper respiratory tract by using the Esmarch manoeuvre	436	5.00	2.00	4.66	1.53
**17**	being capable of ventilating a patient with a face mask (may be using a supraglottic airway tube)	437	5.00	3.00	4.29	1.68
**18**	knowing how to correctly insert a laryngeal mask airway and checking for its correct positioning	437	3.00	2.00	2.90	1.46
**19**	knowing how to correctly insert a laryngeal tube and checking for its correct positioning	438	2.00	3.00	2.66	1.53
**20**	intubating a patient and checking for the correct endotracheal positioning	437	2.00	2.00	2.06	1.13
**21**	performing the initial steps of an emergency algorithm when encountering an unexpected difficult airway	439	2.00	2.00	2.56	1.49
**22**	setting up an adequate mechanical ventilation according to the patient and the operation	437	2.00	2.00	2.06	1.18
	**regional anesthesia and pain therapy**					
**23**	taking patient history focused on pain symptoms	427	4.00	2.00	3.89	1.52
**24**	setting up a therapy plan according to the WHO analgesic ladder	425	4.00	3.00	3.88	1.54
**25**	being accustomed to the usage of patient-controlled anesthesia devices (PCA)	426	2.00	2.00	2.05	1.16
**26**	performing spinal anesthesia	426	1.00	1.00	1.41	0.82
**27**	performing epidural anesthesia	424	1.00	0.00	1.26	0.69

**28**	performing combined spinal/epidural anesthesia (CSE)	421	1.00	0.00	1.18	0.59
	accomplishing a peripheral nerve block by…					
**29**	an interscalene approach to the brachial plexus	428	1.00	0.00	1.15	0.51
**30**	a supraclavicular approach to the brachial plexus	427	1.00	0.00	1.14	0.46
**31**	an axillary approach to the brachial plexus	428	1.00	0.00	1.18	0.57
**32**	blocking the femoral nerve	427	1.00	0.00	1.21	0.60
**33**	blocking the sciatic nerve with a proximal approach	427	1.00	0.00	1.15	0.49
**34**	blocking the sciatic nerve with distal approach	425	1.00	0.00	1.16	0.50

Table 9.10: Overview of all items asked in the individual categories in the field of intensive care medicine and emergency medicine and the average rating of the participants from 1 ("not relevant at all") to 6 ("very relevant") as median and IQR and mean value and standard deviation

	**category**	**competence**	**n**	**median**	**IQR**	**mean**	**SD**
At the end of undergraduate training, the student, as an active member of the professional team, can safely carry out clinical-practical skills adequately and independently under supervision, in a manner that is respectful of the patient. The student can…							
	**intensive care unit**						
**35**	performing a clinically focused physical exam		416	5.00	2.00	4.88	1.34
**36**	transferring information regarding a patient among healthcare professionals utilizing a defined technique (for example SBAR)		415	5.00	3.00	4.55	1.48
**37**	managing an analgosedation for an intervention		411	2.00	2.00	2.21	1.18
**38**	assessing the depth of sedation of a patient using an established scoring system		415	3.00	2.00	2.83	1.50
**39**	demonstrating ultrasound examination utilizing the eFAST principle		418	3.00	3.00	2.77	1.49
**40**	inserting a gastric tube		417	3.00	3.00	3.60	1.62
**41**	inserting a urinary catheter		416	3.00	3.00	3.57	1.69
**42**	performing a bronchoscopy on an intubated patient		418	1.00	1.00	1.43	0.83
**43**	evacuating air by puncturing of a tension pneumothorax		416	3.00	3.00	2.90	1.72

**44**	puncturing and/or drainage of intrapleural fluids	417	2.00	2.00	2.04	1.20
**45**	performing an minithoracotomy and placing a chest tube	415	1.00	1.00	1.72	1.07
**46**	obtaining blood samples for microbiological examination	417	5.00	2.00	4.59	1.57
**47**	performing a blood transfusion according to current guidelines	418	5.00	3.00	4.52	1.73
**48**	calling a patient's death	417	5.00	3.00	4.51	1.75
**49**	inspecting a corpse externally	417	4.00	4.00	4.00	1.83
**50**	completing a death certificate and correctly differentiating the cause of death	415	4.00	4.00	3.73	1.85
**51**	communication adequately with patients and/or relatives in crisis situations	416	4.00	3.00	3.88	1.70
	**emergency medicine**					
**52**	calculating the Glasgow Coma Scale	414	6.00	2.00	5.07	1.29
**53**	evaluating a patient using the ABCDE system	410	6.00	2.00	5.00	1.31
**54**	performing a rapid sequence induction and intubation	414	1.50	1.00	1.87	1.11
**55**	establishing an intraosseous needle	411	2.00	3.00	2.76	1.57
**56**	bringing a patient into lateral recumbent position	412	6.00	1.00	5.41	1.18
**57**	stabilizing the cervical vertebrae using a stifneck	414	5.00	2.00	4.69	1.53
**58**	immobilizing a patient using a vacuum mattress or spineboard	414	4.00	3.00	3.73	1.62
**59**	placing a pelvic binder	413	4.00	3.00	3.45	1.66
**60**	placing a tourniquet	412	4.00	3.00	3.80	1.70
**61**	diagnosing a cardiac arrest	413	6.00	0.00	5.45	1.19
**62**	utilizing the basic life support algorithms according to current guidelines and performing effective chest compressions	412	6.00	0.00	5.51	1.05
	utilizing the advanced life support algorithms according to current guidelines and…					
**63**	correctly analysing the different rhythms in cardiac arrest	414	6.00	2.00	4.89	1.44
**64**	correctly perform defibrillation/cardioversion	414	6.00	2.00	4.87	1.47
**65**	correctly administer drugs	414	6.00	2.00	4.82	1.50

10. Hospital with special supply

The following table shows the rating of the individual learning objectives by the participating physicians working in hospitals with a special supply.

Table 10.1: Overview of all items asked in the individual categories in the field of anesthesiology and the average rating of the participants from 1 ("not relevant at all") to 6 ("very relevant") as median and IQR and mean value and standard deviation

item	category	competence	n	median	IQR	mean	SD
At the end of undergraduate training, the student, as an active member of the professional team, can safely carry out clinical-practical skills adequately and independently under supervision, in a manner that is respectful of the patient. The student can…							
	**premedication visit**						
**1**	taking patient history relevant to anesthesia		80	3.00	2.00	3.18	1.51
**2**	performing an anesthesia focused physical examination (auscultation of heart/lung, status of teeth, predictors of a difficult airway, …)		79	4.00	3.00	3.58	1.46
**3**	performing a 12-channel-ecg and interpretation of the result		80	5.00	3.00	4.40	1.45
**4**	conducting an informed consent discussion with an ASAI/ASAII patient undergoing a low to medium risk operation and documenting it in a legally correct manner		78	2.00	2.00	2.14	1.29
	**preparation of general anesthesia**						
**5**	performing a quick check of the anesthesia working place according to the recommendations of DGAI		73	2.00	2.00	2.03	1.32
**6**	increasing patient safety by completing a standardized preoperative check list (e.g. WHO check list)		72	4.00	3.00	3.93	1.68
**7**	establishing intraoperative monitoring (ecg, non-invasive blood pressure monitoring, temperature, relaxometry, pulse oximetry/oxygen saturation)		73	4.00	3.00	4.23	1.50
**8**	setting up an iv-drip for infusion		73	6.00	2.00	4.86	1.47
**9**	preparing drugs for intravenous application		73	6.00	2.00	4.95	1.31
**10**	establishing a peripheral iv catheter		73	6.00	2.00	4.95	1.47

**11**	establishing a central iv catheter	73	1.00	1.00	1.70	1.04
**12**	establishing an arterial catheter	71	1.00	1.00	1.56	0.98
**13**	applying drugs intravenously, intramuscularly, subcutaneously	72	5.00	2.75	4.63	1.60
	**general anesthesia**					
**14**	being capable of a sufficient preoxygenation	71	4.00	3.00	3.66	1.52
**15**	being able to induce a general anesthesia using hypnotics. opioids and muscle relaxants with adequate dosing	71	1.00	2.00	2.01	1.37
**16**	being able to open the upper respiratory tract by using the Esmarch manoeuvre	71	5.00	3.00	4.52	1.56
**17**	being capable of ventilating a patient with a face mask (may be using a supraglottic airway tube)	71	4.00	4.00	3.96	1.74
**18**	knowing how to correctly insert a laryngeal mask airway and checking for its correct positioning	69	3.00	3.00	3.07	1.76
**19**	knowing how to correctly insert a laryngeal tube and checking for its correct positioning	69	2.00	3.00	2.70	1.65
**20**	intubating a patient and checking for the correct endotracheal positioning	70	2.00	2.00	2.23	1.35
**21**	performing the initial steps of an emergency algorithm when encountering an unexpected difficult airway	70	2.00	2.00	2.31	1.44
**22**	setting up an adequate mechanical ventilation according to the patient and the operation	70	2.00	2.00	1.93	1.13
	**regional anesthesia and pain therapy**					
**23**	taking patient history focused on pain symptoms	69	4.00	2.00	3.71	1.48
**24**	setting up a therapy plan according to the WHO analgesic ladder	69	4.00	3.00	3.72	1.52
**25**	being accustomed to the usage of patient-controlled anesthesia devices (PCA)	69	2.00	1.50	2.01	1.18
**26**	performing spinal anesthesia	69	1.00	0.50	1.38	0.88
**27**	performing epidural anesthesia	69	1.00	0.00	1.29	0.75

**28**	performing combined spinal/epidural anesthesia (CSE)	68	1.00	0.00	1.19	0.72
	accomplishing a peripheral nerve block by…					
**29**	an interscalene approach to the brachial plexus	69	1.00	0.00	1.22	0.72
**30**	a supraclavicular approach to the brachial plexus	69	1.00	0.00	1.16	0.58
**31**	an axillary approach to the brachial plexus	69	1.00	0.00	1.29	0.86
**32**	blocking the femoral nerve	69	1.00	0.50	1.38	0.88
**33**	blocking the sciatic nerve with a proximal approach	69	1.00	0.00	1.22	0.66
**34**	blocking the sciatic nerve with distal approach	68	1.00	0.00	1.22	0.81

Table 10.11: Overview of all items asked in the individual categories in the field of intensive care medicine and emergency medicine and the average rating of the participants from 1 ("not relevant at all") to 6 ("very relevant") as median and IQR and mean value and standard deviation

	**category**	**competence**	**n**	**median**	**IQR**	**mean**	**SD**
At the end of undergraduate training, the student, as an active member of the professional team, can safely carry out clinical-practical skills adequately and independently under supervision, in a manner that is respectful of the patient. The student can…							
	**intensive care unit**						
**35**	performing a clinically focused physical exam		64	5.00	3.00	4.64	1.41
**36**	transferring information regarding a patient among healthcare professionals utilizing a defined technique (for example SBAR)		65	5.00	3.00	4.22	1.58
**37**	managing an analgosedation for an intervention		66	2.00	2.00	2.39	1.49
**38**	assessing the depth of sedation of a patient using an established scoring system		65	3.00	2.00	3.03	1.63
**39**	demonstrating ultrasound examination utilizing the eFAST principle		66	2.00	3.00	2.53	1.63
**40**	inserting a gastric tube		66	3.00	3.00	3.50	1.58
**41**	inserting a urinary catheter		66	3.00	3.00	3.56	1.78
**42**	performing a bronchoscopy on an intubated patient		66	1.00	1.00	1.52	1.01
**43**	evacuating air by puncturing of a tension pneumothorax		66	3.00	4.00	3.18	1.92

**44**	puncturing and/or drainage of intrapleural fluids	65	2.00	2.00	2.06	1.18
**45**	performing an minithoracotomy and placing a chest tube	65	1.00	1.00	1.65	1.11
**46**	obtaining blood samples for microbiological examination	65	5.00	3.00	4.49	1.54
**47**	performing a blood transfusion according to current guidelines	65	5.00	4.00	4.15	1.90
**48**	calling a patient's death	64	5.00	3.00	4.22	1.79
**49**	inspecting a corpse externally	65	4.00	4.00	3.72	1.92
**50**	completing a death certificate and correctly differentiating the cause of death	65	3.00	4.50	3.51	1.97
**51**	communication adequately with patients and/or relatives in crisis situations	65	4.00	3.00	3.58	1.77
	**emergency medicine**					
**52**	calculating the Glasgow Coma Scale	66	6.00	3.00	4.82	1.41
**53**	evaluating a patient using the ABCDE system	64	5.00	3.00	4.39	1.65
**54**	performing a rapid sequence induction and intubation	66	1.00	1.25	2.06	1.51
**55**	establishing an intraosseous needle	66	2.00	3.00	2.77	1.70
**56**	bringing a patient into lateral recumbent position	66	6.00	1.00	5.38	1.22
**57**	stabilizing the cervical vertebrae using a stifneck	64	6.00	1.00	4.78	1.53
**58**	immobilizing a patient using a vacuum mattress or spineboard	65	4.00	2.00	3.77	1.72
**59**	placing a pelvic binder	65	3.00	3.00	2.98	1.71
**60**	placing a tourniquet	65	4.00	2.00	3.78	1.72
**61**	diagnosing a cardiac arrest	65	6.00	0.00	5.51	1.13
**62**	utilizing the basic life support algorithms according to current guidelines and performing effective chest compressions	65	6.00	1.00	5.37	1.18
	utilizing the advanced life support algorithms according to current guidelines and…					
**63**	correctly analysing the different rhythms in cardiac arrest	65	5.00	3.00	4.46	1.71
**64**	correctly perform defibrillation/cardioversion	65	5.00	3.00	4.52	1.72
**65**	correctly administer drugs	65	5.00	3.00	4.42	1.83

11. Rehabilitation hospital

The following table shows the rating of the individual learning objectives by the participating physicians working in rehabilitation hospitals.

Table 11.1: Overview of all items asked in the individual categories in the field of anesthesiology and the average rating of the participants from 1 ("not relevant at all") to 6 ("very relevant") as median and IQR and mean value and standard deviation

item	category	competence	n	median	IQR	mean	SD
At the end of undergraduate training, the student, as an active member of the professional team, can safely carry out clinical-practical skills adequately and independently under supervision, in a manner that is respectful of the patient. The student can…							
	**premedication visit**						
**1**	taking patient history relevant to anesthesia		7	4.00	4.00	4.00	1.73
**2**	performing an anesthesia focused physical examination (auscultation of heart/lung, status of teeth, predictors of a difficult airway, …)		7	4.00	3.00	4.14	1.57
**3**	performing a 12-channel-ecg and interpretation of the result		7	6.00	2.00	5.43	1.13
**4**	conducting an informed consent discussion with an ASAI/ASAII patient undergoing a low to medium risk operation and documenting it in a legally correct manner		7	3.00	3.00	3.14	1.77
	**preparation of general anesthesia**						
**5**	performing a quick check of the anesthesia working place according to the recommendations of DGAI		6	1.50	3.00	2.17	1.94
**6**	increasing patient safety by completing a standardized preoperative check list (e.g. WHO check list)		6	6.00	2.00	5.33	1.03
**7**	establishing intraoperative monitoring (ecg, non-invasive blood pressure monitoring, temperature, relaxometry, pulse oximetry/oxygen saturation)		6	5.00	2.25	4.83	1.17
**8**	setting up an iv-drip for infusion		6	6.00	1.00	5.67	0.52
**9**	preparing drugs for intravenous application		6	6.00	1.00	5.67	0.52

**10**	establishing a peripheral iv catheter	6	6.00	0.25	5.83	0.41
**11**	establishing a central iv catheter	6	2.00	2.25	2.17	1.17
**12**	establishing an arterial catheter	6	2.00	0.25	1.83	0.41
**13**	applying drugs intravenously, intramuscularly, subcutaneously	6	4.50	3.00	4.50	1.38
	**general anesthesia**					
**14**	being capable of a sufficient preoxygenation	6	4.50	2.25	4.67	1.21
**15**	being able to induce a general anesthesia using hypnotics. opioids and muscle relaxants with adequate dosing	6	2.50	2.25	2.33	1.21
**16**	being able to open the upper respiratory tract by using the Esmarch manoeuvre	6	5.50	3.00	4.83	1.47
**17**	being capable of ventilating a patient with a face mask (may be using a supraglottic airway tube)	5	4.00	4.00	4.00	2.00
**18**	knowing how to correctly insert a laryngeal mask airway and checking for its correct positioning	6	3.50	3.25	3.67	1.63
**19**	knowing how to correctly insert a laryngeal tube and checking for its correct positioning	6	4.00	3.50	4.00	2.00
**20**	intubating a patient and checking for the correct endotracheal positioning	6	1.50	1.75	2.00	1.55
**21**	performing the initial steps of an emergency algorithm when encountering an unexpected difficult airway	6	2.00	2.75	2.50	1.87
**22**	setting up an adequate mechanical ventilation according to the patient and the operation	6	2.00	1.25	1.83	0.75
	**regional anesthesia and pain therapy**					
**23**	taking patient history focused on pain symptoms	6	3.50	1.75	3.67	1.37
**24**	setting up a therapy plan according to the WHO analgesic ladder	6	4.00	3.25	4.17	1.60
**25**	being accustomed to the usage of patient-controlled anesthesia devices (PCA)	6	2.00	1.25	2.17	0.75

**26**	performing spinal anesthesia	6	1.00	0.25	1.17	0.41
**27**	performing epidural anesthesia	6	1.00	0.00	1.00	0.00
**28**	performing combined spinal/epidural anesthesia (CSE)	6	1.00	0.00	1.00	0.00
	accomplishing a peripheral nerve block by…					
**29**	an interscalene approach to the brachial plexus	6	1.00	0.00	1.00	0.00
**30**	a supraclavicular approach to the brachial plexus	6	1.00	0.00	1.00	0.00
**31**	an axillary approach to the brachial plexus	6	1.00	0.00	1.00	0.00
**32**	blocking the femoral nerve	6	1.00	0.00	1.00	0.00
**33**	blocking the sciatic nerve with a proximal approach	6	1.00	0.00	1.00	0.00
**34**	blocking the sciatic nerve with distal approach	6	1.00	0.00	1.00	0.00

Table 11.12: Overview of all items asked in the individual categories in the field of intensive care medicine and emergency medicine and the average rating of the participants from 1 ("not relevant at all") to 6 ("very relevant") as median and IQR and mean value and standard deviation

	**category**	**competence**	**n**	**median**	**IQR**	**mean**	**SD**
At the end of undergraduate training, the student, as an active member of the professional team, can safely carry out clinical-practical skills adequately and independently under supervision, in a manner that is respectful of the patient. The student can…							
	**intensive care unit**						
**35**	performing a clinically focused physical exam		6	6.00	1.50	5.33	1.21
**36**	transferring information regarding a patient among healthcare professionals utilizing a defined technique (for example SBAR)		6	4.50	3.00	4.50	1.38
**37**	managing an analgosedation for an intervention		6	2.00	2.50	2.33	1.51
**38**	assessing the depth of sedation of a patient using an established scoring system		6	2.50	2.00	2.83	1.72
**39**	demonstrating ultrasound examination utilizing the eFAST principle		6	1.50	3.25	2.33	1.75
**40**	inserting a gastric tube		6	3.00	3.25	3.83	1.72
**41**	inserting a urinary catheter		6	3.50	4.00	3.83	2.04

**42**	performing a bronchoscopy on an intubated patient	6	1.00	1.00	1.33	0.52
**43**	evacuating air by puncturing of a tension pneumothorax	6	2.50	3.50	2.83	1.94
**44**	puncturing and/or drainage of intrapleural fluids	6	1.00	1.00	1.33	0.52
**45**	performing an minithoracotomy and placing a chest tube	6	1.00	1.25	1.50	0.84
**46**	obtaining blood samples for microbiological examination	6	6.00	2.26	5.17	1.33
**47**	performing a blood transfusion according to current guidelines	6	2.50	2.50	3.17	1.60
**48**	calling a patient's death	6	5.00	3.25	4.50	1.76
**49**	inspecting a corpse externally	6	5.00	3.50	4.33	2.07
**50**	completing a death certificate and correctly differentiating the cause of death	6	5.00	3.50	4.33	2.07
**51**	communication adequately with patients and/or relatives in crisis situations	6	4.00	3.00	4.33	1.51
	**emergency medicine**					
**52**	calculating the Glasgow Coma Scale	6	6.00	2.25	5.17	1.33
**53**	evaluating a patient using the ABCDE system	6	5.00	2.75	4.50	1.87
**54**	performing a rapid sequence induction and intubation	6	1.00	2.00	2.00	2.00
**55**	establishing an intraosseous needle	6	1.00	2.00	2.00	2.00
**56**	bringing a patient into lateral recumbent position	6	6.00	0.25	5.83	0.41
**57**	stabilizing the cervical vertebrae using a stifneck	6	6.00	3.00	5.00	1.55
**58**	immobilizing a patient using a vacuum mattress or spineboard	6	6.00	4.25	4.50	2.35
**59**	placing a pelvic binder	6	5.50	4.25	4.33	2.25
**60**	placing a tourniquet	6	6.00	4.25	4.50	2.35
**61**	diagnosing a cardiac arrest	6	6.00	0.50	5.67	0.82
**62**	utilizing the basic life support algorithms according to current guidelines and performing effective chest compressions	6	6.00	0.75	5.50	1.22
	utilizing the advanced life support algorithms according to current guidelines and…					
**63**	correctly analysing the different rhythms in cardiac arrest	6	5.50	2.25	5.00	1.26
**64**	correctly perform defibrillation/cardioversion	6	4.00	4.00	4.00	1.79
**65**	correctly administer drugs	6	5.00	2.50	4.67	1.63

12. Outpatient anesthesia practice

The following table shows the rating of the individual learning objectives by the participating physicians working in outpatient anesthesia practice.

Table 12.1: Overview of all items asked in the individual categories in the field of anesthesiology and the average rating of the participants from 1 ("not relevant at all") to 6 ("very relevant") as median and IQR and mean value and standard deviation

item	category	competence	n	median	IQR	mean	SD
At the end of undergraduate training, the student, as an active member of the professional team, can safely carry out clinical-practical skills adequately and independently under supervision, in a manner that is respectful of the patient. The student can…							
	**premedication visit**						
**1**	taking patient history relevant to anesthesia		203	3.00	2.00	3.53	1.42
**2**	performing an anesthesia focused physical examination (auscultation of heart/lung, status of teeth, predictors of a difficult airway, …)		200	4.00	2.00	3.97	1.40
**3**	performing a 12-channel-ecg and interpretation of the result		205	4.00	3.00	4.29	1.42
**4**	conducting an informed consent discussion with an ASAI/ASAII patient undergoing a low to medium risk operation and documenting it in a legally correct manner		204	3.00	2.00	2.83	1.46
	**preparation of general anesthesia**						
**5**	performing a quick check of the anesthesia working place according to the recommendations of DGAI		192	2.00	2.00	2.20	1.46
**6**	increasing patient safety by completing a standardized preoperative check list (e.g. WHO check list)		190	4.00	2.00	3.86	1.64
**7**	establishing intraoperative monitoring (ecg, non-invasive blood pressure monitoring, temperature, relaxometry, pulse oximetry/oxygen saturation)		190	4.00	3.00	4.18	1.48
**8**	setting up an iv-drip for infusion		190	6.00	1.25	5.16	1.27
**9**	preparing drugs for intravenous application		189	6.00	2.00	4.95	1.40
**10**	establishing a peripheral iv catheter		192	6.00	1.00	5.14	1.15

**11**	establishing a central iv catheter	191	1.00	1.00	1.79	1.05
**12**	establishing an arterial catheter	191	1.00	1.00	1.65	0.94
**13**	applying drugs intravenously, intramuscularly, subcutaneously	193	5.00	3.00	4.62	1.53
	**general anesthesia**					
**14**	being capable of a sufficient preoxygenation	185	4.00	2.00	3.90	1.57
**15**	being able to induce a general anesthesia using hypnotics. opioids and muscle relaxants with adequate dosing	182	2.00	2.00	2.09	1.26
**16**	being able to open the upper respiratory tract by using the Esmarch manoeuvre	185	6.00	2.00	4.86	1.50
**17**	being capable of ventilating a patient with a face mask (may be using a supraglottic airway tube)	185	4.00	3.00	4.32	1.55
**18**	knowing how to correctly insert a laryngeal mask airway and checking for its correct positioning	185	3.00	2.00	3.02	1.49
**19**	knowing how to correctly insert a laryngeal tube and checking for its correct positioning	183	3.00	2.00	3.11	1.62
**20**	intubating a patient and checking for the correct endotracheal positioning	185	2.00	2.00	2.39	1.34
**21**	performing the initial steps of an emergency algorithm when encountering an unexpected difficult airway	185	2.00	3.00	2.74	1.62
**22**	setting up an adequate mechanical ventilation according to the patient and the operation	185	2.00	2.00	1.96	1.21
	**regional anesthesia and pain therapy**					
**23**	taking patient history focused on pain symptoms	180	4.00	2.00	3.84	1.44
**24**	setting up a therapy plan according to the WHO analgesic ladder	177	3.00	2.00	3.36	1.32
**25**	being accustomed to the usage of patient-controlled anesthesia devices (PCA)	176	2.00	2.00	2.10	1.20
**26**	performing spinal anesthesia	179	1.00	0.00	1.35	0.75
**27**	performing epidural anesthesia	177	1.00	0.00	1.26	0.67

**28**	performing combined spinal/epidural anesthesia (CSE)	177	1.00	0.00	1.24	0.73
	accomplishing a peripheral nerve block by…					
**29**	an interscalene approach to the brachial plexus	180	1.00	0.00	1.22	0.59
**30**	a supraclavicular approach to the brachial plexus	180	1.00	0.00	1.19	0.55
**31**	an axillary approach to the brachial plexus	178	1.00	0.00	1.30	0.75
**32**	blocking the femoral nerve	178	1.00	0.00	1.33	0.75
**33**	blocking the sciatic nerve with a proximal approach	178	1.00	0.00	1.24	0.64
**34**	blocking the sciatic nerve with distal approach	177	1.00	0.00	1.25	0.62

Table 12.13: Overview of all items asked in the individual categories in the field of intensive care medicine and emergency medicine and the average rating of the participants from 1 ("not relevant at all") to 6 ("very relevant") as median and IQR and mean value and standard deviation

	**category**	**competence**	**n**	**median**	**IQR**	**mean**	**SD**
At the end of undergraduate training, the student, as an active member of the professional team, can safely carry out clinical-practical skills adequately and independently under supervision, in a manner that is respectful of the patient. The student can…							
	**intensive care unit**						
**35**	performing a clinically focused physical exam		174	5.00	3.00	4.36	1.64
**36**	transferring information regarding a patient among healthcare professionals utilizing a defined technique (for example SBAR)		172	4.00	3.00	3.65	1.65
**37**	managing an analgosedation for an intervention		174	2.00	2.00	2.14	1.20
**38**	assessing the depth of sedation of a patient using an established scoring system		174	2.00	2.00	2.65	1.43
**39**	demonstrating ultrasound examination utilizing the eFAST principle		175	2.00	1.00	1.97	1.25
**40**	inserting a gastric tube		176	3.00	3.00	3.27	1.65
**41**	inserting a urinary catheter		174	3.00	3.00	3.43	1.65
**42**	performing a bronchoscopy on an intubated patient		174	1.00	1.00	1.46	0.91

**43**	evacuating air by puncturing of a tension pneumothorax	176	2.00	3.00	2.65	1.67
**44**	puncturing and/or drainage of intrapleural fluids	176	2.00	2.00	2.05	1.21
**45**	performing an minithoracotomy and placing a chest tube	175	1.00	1.00	1.81	1.26
**46**	obtaining blood samples for microbiological examination	173	4.00	3.00	4.13	1.67
**47**	performing a blood transfusion according to current guidelines	176	4.00	4.00	3.96	1.82
**48**	calling a patient's death	175	5.00	3.00	4.27	1.78
**49**	inspecting a corpse externally	176	3.00	4.00	3.64	1.92
**50**	completing a death certificate and correctly differentiating the cause of death	176	3.00	3.00	3.44	1.93
**51**	communication adequately with patients and/or relatives in crisis situations	176	4.00	4.00	3.64	1.87
	**emergency medicine**					
**52**	calculating the Glasgow Coma Scale	170	5.00	3.00	4.48	1.60
**53**	evaluating a patient using the ABCDE system	165	5.00	3.00	4.38	1.60
**54**	performing a rapid sequence induction and intubation	170	2.00	2.00	2.03	1.33
**55**	establishing an intraosseous needle	167	2.00	3.00	2.65	1.61
**56**	bringing a patient into lateral recumbent position	170	6.00	0.00	5.59	0.89
**57**	stabilizing the cervical vertebrae using a stifneck	170	6.00	2.00	4.88	1.42
**58**	immobilizing a patient using a vacuum mattress or spineboard	170	5.00	3.00	4.31	1.69
**59**	placing a pelvic binder	170	4.00	2.25	3.66	1.71
**60**	placing a tourniquet	170	4.00	3.00	4.21	1.67
**61**	diagnosing a cardiac arrest	169	6.00	0.50	5.47	1.12
**62**	utilizing the basic life support algorithms according to current guidelines and performing effective chest compressions	169	6.00	1.00	5.33	1.20
	utilizing the advanced life support algorithms according to current guidelines and…					
**63**	correctly analysing the different rhythms in cardiac arrest	168	5.00	3.00	4.46	1.51
**64**	correctly perform defibrillation/cardioversion	167	5.00	3.00	4.54	1.52
**65**	correctly administer drugs	168	5.00	3.00	4.45	1.56

13. Additional certification in emergency medicine

The following table shows the rating of the individual learning objectives by the participating physicians with an additional certification in emergency medicine.

Table 13.1: Overview of all items asked in the individual categories in the field of anesthesiology and the average rating of the participants from 1 ("not relevant at all") to 6 ("very relevant") as median and IQR and mean value and standard deviation

item	category	competence	n	median	IQR	mean	SD
At the end of undergraduate training, the student, as an active member of the professional team, can safely carry out clinical-practical skills adequately and independently under supervision, in a manner that is respectful of the patient. The student can…							
	**premedication visit**						
**1**	taking patient history relevant to anesthesia		1695	3.00	2.00	3.26	1.40
**2**	performing an anesthesia focused physical examination (auscultation of heart/lung, status of teeth, predictors of a difficult airway, …)		1652	4.00	2.00	3.73	1.43
**3**	performing a 12-channel-ecg and interpretation of the result		1676	5.00	2.00	4.63	1.38
**4**	conducting an informed consent discussion with an ASAI/ASAII patient undergoing a low to medium risk operation and documenting it in a legally correct manner		1684	2.00	2.00	2.51	1.38
	**preparation of general anesthesia**						
**5**	performing a quick check of the anesthesia working place according to the recommendations of DGAI		1591	1.00	1.00	1.96	1.33
**6**	increasing patient safety by completing a standardized preoperative check list (e.g. WHO check list)		1575	4.00	3.00	4.08	1.66
**7**	establishing intraoperative monitoring (ecg, non-invasive blood pressure monitoring, temperature, relaxometry, pulse oximetry/oxygen saturation)		1576	4.00	3.00	4.25	1.49
**8**	setting up an iv-drip for infusion		1576	6.00	2.00	5.06	1.32
**9**	preparing drugs for intravenous application		1568	6.00	2.00	4.95	1.37
**10**	establishing a peripheral iv catheter		1572	6.00	2.00	5.09	1.25

**11**	establishing a central iv catheter	1577	2.00	1.00	1.85	1.08
**12**	establishing an arterial catheter	1555	1.00	1.00	1.82	1.08
**13**	applying drugs intravenously, intramuscularly, subcutaneously	1579	5.00	3.00	4.55	1.51
	**general anesthesia**					
**14**	being capable of a sufficient preoxygenation	1523	4.00	3.00	3.59	1.65
**15**	being able to induce a general anesthesia using hypnotics. opioids and muscle relaxants with adequate dosing	1517	2.00	2.00	1.95	1.16
**16**	being able to open the upper respiratory tract by using the Esmarch manoeuvre	1528	5.00	3.00	4.55	1.59
**17**	being capable of ventilating a patient with a face mask (may be using a supraglottic airway tube)	1524	4.00	3.00	4.10	1.69
**18**	knowing how to correctly insert a laryngeal mask airway and checking for its correct positioning	1527	3.00	2.00	2.81	1.54
**19**	knowing how to correctly insert a laryngeal tube and checking for its correct positioning	1520	2.00	3.00	2.69	1.59
**20**	intubating a patient and checking for the correct endotracheal positioning	1520	2.00	2.00	2.06	1.22
**21**	performing the initial steps of an emergency algorithm when encountering an unexpected difficult airway	1527	2.00	2.00	2.40	1.51
**22**	setting up an adequate mechanical ventilation according to the patient and the operation	1519	1.00	1.00	1.87	1.12
	**regional anesthesia and pain therapy**					
**23**	taking patient history focused on pain symptoms	1497	4.00	2.00	3.71	1.49
**24**	setting up a therapy plan according to the WHO analgesic ladder	1483	4.00	3.00	3.62	1.50
**25**	being accustomed to the usage of patient-controlled anesthesia devices (PCA)	1486	2.00	2.00	1.94	1.14
**26**	performing spinal anesthesia	1489	1.00	0.00	1.33	0.75
**27**	performing epidural anesthesia	1487	1.00	0.00	1.22	0.62

**28**	performing combined spinal/epidural anesthesia (CSE)	1477	1.00	0.00	1.15	0.54
	accomplishing a peripheral nerve block by…					
**29**	an interscalene approach to the brachial plexus	1495	1.00	0.00	1.15	0.53
**30**	a supraclavicular approach to the brachial plexus	1491	1.00	0.00	1.13	0.49
**31**	an axillary approach to the brachial plexus	1494	1.00	0.00	1.19	0.62
**32**	blocking the femoral nerve	1491	1.00	0.00	1.21	0.64
**33**	blocking the sciatic nerve with a proximal approach	1491	1.00	0.00	1.15	0.53
**34**	blocking the sciatic nerve with distal approach	1479	1.00	0.00	1.16	0.54

Table 13.14: Overview of all items asked in the individual categories in the field of intensive care medicine and emergency medicine and the average rating of the participants from 1 ("not relevant at all") to 6 ("very relevant") as median and IQR and mean value and standard deviation

	**category**	**competence**	**n**	**median**	**IQR**	**mean**	**SD**
At the end of undergraduate training, the student, as an active member of the professional team, can safely carry out clinical-practical skills adequately and independently under supervision, in a manner that is respectful of the patient. The student can…							
	**intensive care unit**						
**35**	performing a clinically focused physical exam		1459	5.00	2.00	4.70	1.41
**36**	transferring information regarding a patient among healthcare professionals utilizing a defined technique (for example SBAR)		1449	4.00	3.00	4.10	1.63
**37**	managing an analgosedation for an intervention		1452	2.00	2.00	2.16	1.22
**38**	assessing the depth of sedation of a patient using an established scoring system		1456	2.00	3.00	2.68	1.47
**39**	demonstrating ultrasound examination utilizing the eFAST principle		1460	2.00	2.00	2.47	1.48
**40**	inserting a gastric tube		1460	3.00	3.00	3.49	1.61
**41**	inserting a urinary catheter		1457	3.00	3.00	3.45	1.70
**42**	performing a bronchoscopy on an intubated patient		1456	1.00	0.00	1.39	0.82
**43**	evacuating air by puncturing of a tension pneumothorax		1456	2.00	3.00	2.84	1.74

**44**	puncturing and/or drainage of intrapleural fluids	1460	2.00	2.00	2.01	1.20
**45**	performing an minithoracotomy and placing a chest tube	1450	1.00	1.00	1.70	1.11
**46**	obtaining blood samples for microbiological examination	1454	5.00	3.00	4.50	1.56
**47**	performing a blood transfusion according to current guidelines	1457	5.00	3.00	4.26	1.78
**48**	calling a patient's death	1457	5.00	3.00	4.49	1.72
**49**	inspecting a corpse externally	1459	4.00	4.00	3.94	1.86
**50**	completing a death certificate and correctly differentiating the cause of death	1453	4.00	4.00	3.75	1.90
**51**	communication adequately with patients and/or relatives in crisis situations	1458	4.00	3.00	3.69	1.74
	**emergency medicine**					
**52**	calculating the Glasgow Coma Scale	1439	6.00	2.00	4.87	1.42
**53**	evaluating a patient using the ABCDE system	1417	5.00	2.00	4.74	1.48
**54**	performing a rapid sequence induction and intubation	1436	1.00	1.00	1.79	1.15
**55**	establishing an intraosseous needle	1433	2.00	3.00	2.64	1.62
**56**	bringing a patient into lateral recumbent position	1436	6.00	1.00	5.42	1.17
**57**	stabilizing the cervical vertebrae using a stifneck	1436	5.00	2.00	4.69	1.56
**58**	immobilizing a patient using a vacuum mattress or spineboard	1438	4.00	4.00	3.93	1.72
**59**	placing a pelvic binder	1434	3.00	3.00	3.40	1.72
**60**	placing a tourniquet	1430	4.00	4.00	3.91	1.73
**61**	diagnosing a cardiac arrest	1436	6.00	0.00	5.44	1.15
**62**	utilizing the basic life support algorithms according to current guidelines and performing effective chest compressions	1436	6.00	1.00	5.40	1.19
	utilizing the advanced life support algorithms according to current guidelines and…					
**63**	correctly analysing the different rhythms in cardiac arrest	1097	5.00	2.00	4.64	1.51
**64**	correctly perform defibrillation/cardioversion	1435	5.00	2.00	4.64	1.58
**65**	correctly administer drugs	1436	5.00	3.00	4.58	1.59

14. Additional certification in intensive care medicine

The following table shows the rating of the individual learning objectives by the participating physicians with an additional certification in intensive care medicine.

Table 14.1: Overview of all items asked in the individual categories in the field of anesthesiology and the average rating of the participants from 1 ("not relevant at all") to 6 ("very relevant") as median and IQR and mean value and standard deviation

item	category	competence	n	median	IQR	mean	SD
At the end of undergraduate training, the student, as an active member of the professional team, can safely carry out clinical-practical skills adequately and independently under supervision, in a manner that is respectful of the patient. The student can…							
	**premedication visit**						
**1**	taking patient history relevant to anesthesia		1097	3.00	2.00	3.26	1.43
**2**	performing an anesthesia focused physical examination (auscultation of heart/lung, status of teeth, predictors of a difficult airway, …)		1068	4.00	2.00	3.71	1.46
**3**	performing a 12-channel-ecg and interpretation of the result		1082	5.00	2.00	4.61	1.40
**4**	conducting an informed consent discussion with an ASAI/ASAII patient undergoing a low to medium risk operation and documenting it in a legally correct manner		1092	2.00	2.00	2.54	1.39
	**preparation of general anesthesia**						
**5**	performing a quick check of the anesthesia working place according to the recommendations of DGAI		1028	1.00	2.00	1.99	1.39
**6**	increasing patient safety by completing a standardized preoperative check list (e.g. WHO check list)		1015	4.00	3.00	4.10	1.68
**7**	establishing intraoperative monitoring (ecg, non-invasive blood pressure monitoring, temperature, relaxometry, pulse oximetry/oxygen saturation)		1019	4.00	2.00	4.11	1.52
**8**	setting up an iv-drip for infusion		1018	6.00	2.00	4.94	1.40
**9**	preparing drugs for intravenous application		1014	6.00	2.00	4.86	1.44
**10**	establishing a peripheral iv catheter		1013	6.00	2.00	5.03	1.27

**11**	establishing a central iv catheter	1018	1.00	1.00	1.81	1.05
**12**	establishing an arterial catheter	1002	1.00	1.00	1.76	1.06
**13**	applying drugs intravenously, intramuscularly, subcutaneously	1019	5.00	3.00	4.52	1.51
	**general anesthesia**					
**14**	being capable of a sufficient preoxygenation	986	3.00	3.00	3.46	1.67
**15**	being able to induce a general anesthesia using hypnotics. opioids and muscle relaxants with adequate dosing	984	2.00	1.00	1.91	1.16
**16**	being able to open the upper respiratory tract by using the Esmarch manoeuvre	989	5.00	3.00	4.48	1.64
**17**	being capable of ventilating a patient with a face mask (may be using a supraglottic airway tube)	985	4.00	3.00	3.98	1.70
**18**	knowing how to correctly insert a laryngeal mask airway and checking for its correct positioning	991	2.00	3.00	2.74	1.54
**19**	knowing how to correctly insert a laryngeal tube and checking for its correct positioning	984	2.00	3.00	2.57	1.55
**20**	intubating a patient and checking for the correct endotracheal positioning	984	2.00	2.00	2.04	1.22
**21**	performing the initial steps of an emergency algorithm when encountering an unexpected difficult airway	990	2.00	2.00	2.33	1.50
**22**	setting up an adequate mechanical ventilation according to the patient and the operation	985	1.00	1.00	1.82	1.12
	**regional anesthesia and pain therapy**					
**23**	taking patient history focused on pain symptoms	978	4.00	2.00	3.68	1.49
**24**	setting up a therapy plan according to the WHO analgesic ladder	969	4.00	3.00	3.60	1.50
**25**	being accustomed to the usage of patient-controlled anesthesia devices (PCA)	968	2.00	2.00	1.96	1.18
**26**	performing spinal anesthesia	973	1.00	0.00	1.33	0.77
**27**	performing epidural anesthesia	973	1.00	0.00	1.21	0.64

**28**	performing combined spinal/epidural anesthesia (CSE)	966	1.00	0.00	1.14	0.54
	accomplishing a peripheral nerve block by…					
**29**	an interscalene approach to the brachial plexus	975	1.00	0.00	1.15	0.56
**30**	a supraclavicular approach to the brachial plexus	972	1.00	0.00	1.13	0.52
**31**	an axillary approach to the brachial plexus	972	1.00	0.00	1.20	0.65
**32**	blocking the femoral nerve	970	1.00	0.00	1.22	0.67
**33**	blocking the sciatic nerve with a proximal approach	971	1.00	0.00	1.14	0.53
**34**	blocking the sciatic nerve with distal approach	961	1.00	0.00	1.16	0.56

Table 14.15: Overview of all items asked in the individual categories in the field of intensive care medicine and emergency medicine and the average rating of the participants from 1 ("not relevant at all") to 6 ("very relevant") as median and IQR and mean value and standard deviation

	category	competence	n	median	IQR	mean	SD
At the end of undergraduate training, the student, as an active member of the professional team, can safely carry out clinical-practical skills adequately and independently under supervision, in a manner that is respectful of the patient. The student can…							
	**intensive care unit**						
**35**	performing a clinically focused physical exam		959	5.00	2.00	4.65	1.44
**36**	transferring information regarding a patient among healthcare professionals utilizing a defined technique (for example SBAR)		952	4.00	3.00	4.01	1.63
**37**	managing an analgosedation for an intervention		951	2.00	2.00	2.08	1.21
**38**	assessing the depth of sedation of a patient using an established scoring system		955	2.00	3.00	2.68	1.49
**39**	demonstrating ultrasound examination utilizing the eFAST principle		959	2.00	2.00	2.47	1.49
**40**	inserting a gastric tube		958	3.00	3.00	3.48	1.64
**41**	inserting a urinary catheter		954	3.00	3.00	3.49	1.71
**42**	performing a bronchoscopy on an intubated patient		956	1.00	0.00	1.39	0.81
**43**	evacuating air by puncturing of a tension pneumothorax		956	2.00	3.00	2.82	1.74

**44**	puncturing and/or drainage of intrapleural fluids	956	2.00	2.00	2.02	1.23
**45**	performing an minithoracotomy and placing a chest tube	953	1.00	1.00	1.67	1.11
**46**	obtaining blood samples for microbiological examination	955	5.00	3.00	4.45	1.60
**47**	performing a blood transfusion according to current guidelines	954	5.00	3.00	4.23	1.83
**48**	calling a patient's death	956	5.00	3.00	4.42	1.79
**49**	inspecting a corpse externally	956	4.00	4.00	3.83	1.92
**50**	completing a death certificate and correctly differentiating the cause of death	954	4.00	4.00	3.69	1.92
**51**	communication adequately with patients and/or relatives in crisis situations	956	4.00	3.00	3.60	1.75
	**emergency medicine**					
**52**	calculating the Glasgow Coma Scale	947	6.00	2.00	4.87	1.44
**53**	evaluating a patient using the ABCDE system	934	5.00	2.00	4.70	1.52
**54**	performing a rapid sequence induction and intubation	945	1.00	1.00	1.71	1.12
**55**	establishing an intraosseous needle	943	2.00	3.00	2.54	1.61
**56**	bringing a patient into lateral recumbent position	943	6.00	1.00	5.40	1.23
**57**	stabilizing the cervical vertebrae using a stifneck	943	5.00	2.00	4.73	1.54
**58**	immobilizing a patient using a vacuum mattress or spineboard	947	4.00	3.00	3.99	1.74
**59**	placing a pelvic binder	942	3.00	3.00	3.34	1.74
**60**	placing a tourniquet	942	4.00	4.00	3.89	1.75
**61**	diagnosing a cardiac arrest	943	6.00	0.00	5.41	1.21
**62**	utilizing the basic life support algorithms according to current guidelines and performing effective chest compressions	943	6.00	1.00	5.35	1.24
	utilizing the advanced life support algorithms according to current guidelines and…					
**63**	correctly analysing the different rhythms in cardiac arrest	942	5.00	3.00	4.56	1.54
**64**	correctly perform defibrillation/cardioversion	942	5.00	3.00	4.58	1.61
**65**	correctly administer drugs	941	5.00	3.00	4.52	1.61

15. Additional certification in pain medicine

The following table shows the rating of the individual learning objectives by the participating physicians with an additional certification in pain medicine.

Table 15.1: Overview of all items asked in the individual categories in the field of anesthesiology and the average rating of the participants from 1 ("not relevant at all") to 6 ("very relevant") as median and IQR and mean value and standard deviation

item	category	competence	n	median	IQR	mean	SD
At the end of undergraduate training, the student, as an active member of the professional team, can safely carry out clinical-practical skills adequately and independently under supervision, in a manner that is respectful of the patient. The student can…							
	**premedication visit**						
**1**	taking patient history relevant to anesthesia		335	3.00	2.00	3.28	1.40
**2**	performing an anesthesia focused physical examination (auscultation of heart/lung, status of teeth, predictors of a difficult airway, …)		326	4.00	2.00	3.78	1.47
**3**	performing a 12-channel-ecg and interpretation of the result		327	5.00	2.00	4.57	1.37
**4**	conducting an informed consent discussion with an ASAI/ASAII patient undergoing a low to medium risk operation and documenting it in a legally correct manner		330	2.00	2.00	2.63	1.35
	**preparation of general anesthesia**						
**5**	performing a quick check of the anesthesia working place according to the recommendations of DGAI		311	1.00	2.00	1.98	1.31
**6**	increasing patient safety by completing a standardized preoperative check list (e.g. WHO check list)		308	4.00	3.00	4.13	1.64
**7**	establishing intraoperative monitoring (ecg, non-invasive blood pressure monitoring, temperature, relaxometry, pulse oximetry/oxygen saturation)		309	4.00	2.00	4.08	1.50
**8**	setting up an iv-drip for infusion		308	6.00	2.00	4.98	1.41
**9**	preparing drugs for intravenous application		308	6.00	2.00	4.91	1.41

**10**	establishing a peripheral iv catheter	307	6.00	2.00	5.06	1.26
**11**	establishing a central iv catheter	307	2.00	1.00	1.84	1.02
**12**	establishing an arterial catheter	303	1.00	1.00	1.78	1.07
**13**	applying drugs intravenously, intramuscularly, subcutaneously	309	5.00	3.00	4.57	1.49
	**general anesthesia**					
**14**	being capable of a sufficient preoxygenation	302	4.00	3.00	3.57	1.65
**15**	being able to induce a general anesthesia using hypnotics. opioids and muscle relaxants with adequate dosing	303	2.00	2.00	1.94	1.12
**16**	being able to open the upper respiratory tract by using the Esmarch manoeuvre	305	5.00	3.00	4.51	1.60
**17**	being capable of ventilating a patient with a face mask (may be using a supraglottic airway tube)	304	4.00	3.00	4.01	1.71
**18**	knowing how to correctly insert a laryngeal mask airway and checking for its correct positioning	305	2.00	2.00	2.75	1.47
**19**	knowing how to correctly insert a laryngeal tube and checking for its correct positioning	304	2.00	3.00	2.69	1.57
**20**	intubating a patient and checking for the correct endotracheal positioning	302	2.00	2.00	2.14	1.25
**21**	performing the initial steps of an emergency algorithm when encountering an unexpected difficult airway	306	2.00	2.00	2.35	1.42
**22**	setting up an adequate mechanical ventilation according to the patient and the operation	304	1.00	1.00	1.80	1.07
	**regional anesthesia and pain therapy**					
**23**	taking patient history focused on pain symptoms	301	4.00	2.50	3.59	1.42
**24**	setting up a therapy plan according to the WHO analgesic ladder	298	4.00	3.00	3.61	1.48
**25**	being accustomed to the usage of patient-controlled anesthesia devices (PCA)	300	2.00	2.00	2.03	1.20
**26**	performing spinal anesthesia	301	1.00	0.00	1.32	0.75

**27**	performing epidural anesthesia	301	1.00	0.00	1.22	0.67
**28**	performing combined spinal/epidural anesthesia (CSE)	299	1.00	0.00	1.15	0.60
	accomplishing a peripheral nerve block by…					
**29**	an interscalene approach to the brachial plexus	302	1.00	0.00	1.11	0.51
**30**	a supraclavicular approach to the brachial plexus	302	1.00	0.00	1.11	0.46
**31**	an axillary approach to the brachial plexus	303	1.00	0.00	1.18	0.64
**32**	blocking the femoral nerve	302	1.00	0.00	1.21	0.67
**33**	blocking the sciatic nerve with a proximal approach	303	1.00	0.00	1.13	0.51
**34**	blocking the sciatic nerve with distal approach	299	1.00	0.00	1.13	0.49

Table 15.16: Overview of all items asked in the individual categories in the field of intensive care medicine and emergency medicine and the average rating of the participants from 1 ("not relevant at all") to 6 ("very relevant") as median and IQR and mean value and standard deviation

	**category**	**competence**	**n**	**median**	**IQR**	**mean**	**SD**
At the end of undergraduate training, the student, as an active member of the professional team, can safely carry out clinical-practical skills adequately and independently under supervision, in a manner that is respectful of the patient. The student can…							
	**intensive care unit**						
**35**	performing a clinically focused physical exam		289	5.00	3.00	4.45	1.48
**36**	transferring information regarding a patient among healthcare professionals utilizing a defined technique (for example SBAR)		288	4.00	2.00	3.89	1.60
**37**	managing an analgosedation for an intervention		289	2.00	2.00	2.12	1.20
**38**	assessing the depth of sedation of a patient using an established scoring system		289	2.00	1.00	2.61	1.40
**39**	demonstrating ultrasound examination utilizing the eFAST principle		291	2.00	2.00	2.33	1.40
**40**	inserting a gastric tube		291	3.00	3.00	3.40	1.68
**41**	inserting a urinary catheter		291	3.00	3.00	3.46	1.71
**42**	performing a bronchoscopy on an intubated patient		288	1.00	1.00	1.39	0.84

**43**	evacuating air by puncturing of a tension pneumothorax	290	2.00	3.00	2.72	1.73
**44**	puncturing and/or drainage of intrapleural fluids	291	2.00	2.00	1.93	1.18
**45**	performing an minithoracotomy and placing a chest tube	291	1.00	1.00	1.61	1.05
**46**	obtaining blood samples for microbiological examination	291	4.00	3.00	4.23	1.64
**47**	performing a blood transfusion according to current guidelines	292	5.00	3.75	4.16	1.81
**48**	calling a patient's death	292	5.00	3.00	4.40	1.80
**49**	inspecting a corpse externally	290	4.00	4.00	3.87	1.91
**50**	completing a death certificate and correctly differentiating the cause of death	288	4.00	4.00	3.71	1.93
**51**	communication adequately with patients and/or relatives in crisis situations	291	4.00	3.00	3.72	1.73
	**emergency medicine**					
**52**	calculating the Glasgow Coma Scale	288	5.00	3.00	4.60	1.57
**53**	evaluating a patient using the ABCDE system	284	5.00	3.00	4.44	1.63
**54**	performing a rapid sequence induction and intubation	288	1.00	1.00	1.74	1.19
**55**	establishing an intraosseous needle	287	2.00	2.00	2.46	1.62
**56**	bringing a patient into lateral recumbent position	290	6.00	1.00	5.35	1.29
**57**	stabilizing the cervical vertebrae using a stifneck	289	6.00	2.00	4.71	1.58
**58**	immobilizing a patient using a vacuum mattress or spineboard	288	4.00	3.00	4.05	1.74
**59**	placing a pelvic binder	290	3.00	3.00	3.32	1.75
**60**	placing a tourniquet	287	4.00	3.00	3.87	1.69
**61**	diagnosing a cardiac arrest	288	6.00	1.00	5.36	1.23
**62**	utilizing the basic life support algorithms according to current guidelines and performing effective chest compressions	288	6.00	1.00	5.21	1.36
	utilizing the advanced life support algorithms according to current guidelines and…					
**63**	correctly analysing the different rhythms in cardiac arrest	286	5.00	3.00	4.43	1.51
**64**	correctly perform defibrillation/cardioversion	285	5.00	3.00	4.42	1.62
**65**	correctly administer drugs	287	5.00	3.00	4.35	1.63

16. Additional certification in palliative care

The following table shows the rating of the individual learning objectives by the participating physicians with an additional certification in palliative care.

Table 16.1: Overview of all items asked in the individual categories in the field of anesthesiology and the average rating of the participants from 1 ("not relevant at all") to 6 ("very relevant") as median and IQR and mean value and standard deviation

item	category	competence	n	median	IQR	mean	SD
At the end of undergraduate training, the student, as an active member of the professional team, can safely carry out clinical-practical skills adequately and independently under supervision, in a manner that is respectful of the patient. The student can…							
	**premedication visit**						
**1**	taking patient history relevant to anesthesia		234	3.00	2.00	3.37	1.49
**2**	performing an anesthesia focused physical examination (auscultation of heart/lung, status of teeth, predictors of a difficult airway, …)		230	4.00	2.00	3.67	1.46
**3**	performing a 12-channel-ecg and interpretation of the result		231	5.00	2.00	4.73	1.34
**4**	conducting an informed consent discussion with an ASAI/ASAII patient undergoing a low to medium risk operation and documenting it in a legally correct manner		231	2.00	2.00	2.65	1.40
	**preparation of general anesthesia**						
**5**	performing a quick check of the anesthesia working place according to the recommendations of DGAI		220	1.00	2.00	1.96	1.32
**6**	increasing patient safety by completing a standardized preoperative check list (e.g. WHO check list)		219	4.00	3.00	4.06	1.67
**7**	establishing intraoperative monitoring (ecg, non-invasive blood pressure monitoring, temperature, relaxometry, pulse oximetry/oxygen saturation)		218	4.00	3.00	4.27	1.55
**8**	setting up an iv-drip for infusion		219	6.00	2.00	5.13	1.33
**9**	preparing drugs for intravenous application		221	6.00	2.00	4.99	1.42
**10**	establishing a peripheral iv catheter		219	6.00	2.00	4.99	1.31

**11**	establishing a central iv catheter	219	2.00	1.00	1.89	1.13
**12**	establishing an arterial catheter	214	1.00	1.00	1.78	1.08
**13**	applying drugs intravenously, intramuscularly, subcutaneously	221	5.00	3.00	4.52	1.59
	**general anesthesia**					
**14**	being capable of a sufficient preoxygenation	211	4.00	3.00	3.62	1.71
**15**	being able to induce a general anesthesia using hypnotics. opioids and muscle relaxants with adequate dosing	211	1.00	1.00	1.92	1.23
**16**	being able to open the upper respiratory tract by using the Esmarch manoeuvre	212	5.00	3.00	4.51	1.64
**17**	being capable of ventilating a patient with a face mask (may be using a supraglottic airway tube)	212	4.00	3.00	4.03	1.69
**18**	knowing how to correctly insert a laryngeal mask airway and checking for its correct positioning	212	2.00	2.00	2.77	1.54
**19**	knowing how to correctly insert a laryngeal tube and checking for its correct positioning	212	2.00	3.00	2.71	1.55
**20**	intubating a patient and checking for the correct endotracheal positioning	210	2.00	2.00	2.16	1.28
**21**	performing the initial steps of an emergency algorithm when encountering an unexpected difficult airway	213	2.00	2.00	2.34	1.47
**22**	setting up an adequate mechanical ventilation according to the patient and the operation	211	1.00	1.00	1.82	1.08
	**regional anesthesia and pain therapy**					
**23**	taking patient history focused on pain symptoms	207	4.00	2.00	3.76	1.53
**24**	setting up a therapy plan according to the WHO analgesic ladder	207	4.00	2.00	3.78	1.47
**25**	being accustomed to the usage of patient-controlled anesthesia devices (PCA)	208	2.00	2.00	2.07	1.17
**26**	performing spinal anesthesia	206	1.00	0.00	1.29	0.68
**27**	performing epidural anesthesia	207	1.00	0.00	1.16	0.52
**28**	performing combined spinal/epidural anesthesia (CSE)	207	1.00	0.00	1.11	0.48

	accomplishing a peripheral nerve block by…					
**29**	an interscalene approach to the brachial plexus	209	1.00	0.00	1.05	0.24
**30**	a supraclavicular approach to the brachial plexus	209	1.00	0.00	1.05	0.24
**31**	an axillary approach to the brachial plexus	208	1.00	0.00	1.13	0.49
**32**	blocking the femoral nerve	208	1.00	0.00	1.16	0.56
**33**	blocking the sciatic nerve with a proximal approach	208	1.00	0.00	1.08	0.32
**34**	blocking the sciatic nerve with distal approach	206	1.00	0.00	1.08	0.31

Table 16.17: Overview of all items asked in the individual categories in the field of intensive care medicine and emergency medicine and the average rating of the participants from 1 ("not relevant at all") to 6 ("very relevant") as median and IQR and mean value and standard deviation

	**category**	**competence**	**n**	**median**	**IQR**	**mean**	**SD**
At the end of undergraduate training, the student, as an active member of the professional team, can safely carry out clinical-practical skills adequately and independently under supervision, in a manner that is respectful of the patient. The student can…							
	**intensive care unit**						
**35**	performing a clinically focused physical exam		204	5.00	2.75	4.64	1.51
**36**	transferring information regarding a patient among healthcare professionals utilizing a defined technique (for example SBAR)		206	4.00	3.00	4.01	1.65
**37**	managing an analgosedation for an intervention		206	2.00	2.00	2.17	1.28
**38**	assessing the depth of sedation of a patient using an established scoring system		206	2.00	2.25	2.78	1.55
**39**	demonstrating ultrasound examination utilizing the eFAST principle		208	2.00	2.75	2.52	1.57
**40**	inserting a gastric tube		207	3.00	3.00	3.60	1.76
**41**	inserting a urinary catheter		208	4.00	3.75	3.61	1.80
**42**	performing a bronchoscopy on an intubated patient		206	1.00	1.00	1.40	0.75
**43**	evacuating air by puncturing of a tension pneumothorax		208	2.00	3.00	2.76	1.68
**44**	puncturing and/or drainage of intrapleural fluids		208	2.00	2.00	2.16	1.26

**45**	performing an minithoracotomy and placing a chest tube	208	1.00	1.00	1.70	1.07
**46**	obtaining blood samples for microbiological examination	207	5.00	3.00	4.48	1.65
**47**	performing a blood transfusion according to current guidelines	208	5.00	3.00	4.28	1.87
**48**	calling a patient's death	208	6.00	3.00	4.71	1.68
**49**	inspecting a corpse externally	205	5.00	3.50	4.17	1.84
**50**	completing a death certificate and correctly differentiating the cause of death	203	4.00	4.00	3.95	1.88
**51**	communication adequately with patients and/or relatives in crisis situations	206	4.00	4.00	3.88	1.79
	**emergency medicine**					
**52**	calculating the Glasgow Coma Scale	203	5.00	2.00	4.67	1.57
**53**	evaluating a patient using the ABCDE system	201	5.00	3.00	4.58	1.60
**54**	performing a rapid sequence induction and intubation	204	1.00	1.00	1.75	1.12
**55**	establishing an intraosseous needle	204	2.00	2.00	2.49	1.63
**56**	bringing a patient into lateral recumbent position	204	6.00	0.00	5.41	1.25
**57**	stabilizing the cervical vertebrae using a stifneck	205	6.00	2.00	4.72	1.56
**58**	immobilizing a patient using a vacuum mattress or spineboard	204	4.00	4.00	3.95	1.76
**59**	placing a pelvic binder	204	3.00	3.00	3.33	1.77
**60**	placing a tourniquet	203	4.00	4.00	3.80	1.79
**61**	diagnosing a cardiac arrest	204	6.00	0.00	5.43	1.18
**62**	utilizing the basic life support algorithms according to current guidelines and performing effective chest compressions	204	6.00	1.00	5.33	1.30
	utilizing the advanced life support algorithms according to current guidelines and…					
**63**	correctly analysing the different rhythms in cardiac arrest	205	5.00	3.00	4.58	1.58
**64**	correctly perform defibrillation/cardioversion	204	5.00	3.00	4.63	1.61
**65**	correctly administer drugs	205	5.00	3.00	4.58	1.61
