# Supplementary material for: Teaching Practical Skills in Anesthesia, Intensive Care, Emergency and Pain Medicine—What Is Really Relevant for Medical Students? Results of a German National Survey of Nearly 3000 Anesthesiologists
Source: Healthcare (Basel). 2022 Nov 11;10(11):2260. doi: 10.3390/healthcare10112260 (PMC9690720; doi:10.3390/healthcare10112260)
Supplement: Supplementary file 1 [file healthcare-10-02260-s001.zip › Table S2.pdf]

Supplement Table S2

The following supplemental tables provide a summary of significant differences in how the various groups of physician respondents rated each learning objective.

Table of contents

1. Significant differences in the rating depending on the level of professional qualification . 2
2. Significant differences in the evaluation depending on the workplace............................12
3. Significant differences in the evaluation depending additional certifications...................19
   a) Physicians with additional certification in emergency medicine 19
   b) Physicians with additional certification in intensive care medicine.............................20
   c) Physicians with additional certification in palliative care 21
   d) Physicians with additional certification in pain medicine 22

1. Significant differences in the rating depending on the level of professional qualification

The following table shows an overview of significant differences in the evaluation of individual learning objectives depending on the professional qualification and position of the surveyed physicians.

Comparison of the different positions						
**item**	**competence**	**position 1**		**position 2**		**p**
		**median (IQR)**	**mean ±SD**	**median (IQR)**	**mean ±SD**	
At the end of undergraduate training, the student, as an active member of the professional team, can safely carry out clinical-practical skills adequately and independently under supervision, in a manner that is respectful of the patient. The student can…						
**3**	performing a 12-channel-ecg and interpretation of the result	**Retired physician**		**Specialist**		
		4.00 (2.00)	4.00 ±1.49	5.00 (2.00)	4.58 ±1.38	<0.001
		**Retired physician**		**Chief physician**		
		4.00 (2.00)	4.00 ±1.49	5.00 (2.00)	4.59 ±1.39	0.001
		**Retired physician**		**Consultant**		
		4.00 (2.00)	4.00 ±1.49	5.00 (2.00)	4.66 ±1.37	<0.001
		**Retired physician**		**Doctors in training**		
		4.00 (2.00)	4.00 ±1.49	5.00 (2.00)	4.89 ±1.27	<0.001
		**Specialist**		**Doctors in training**		
		5.00 (2.00)	4.58 ±1.38	5.00 (2.00)	4.89 ±1.27	0.007
**6**	increasing patient safety by completing a standardized preoperative check list (e.g. WHO check list)	**Doctors in training**		**Consultant**		
		4.00 (2.00)	3.82 ±1.64	4.00 (3.00)	4.16 ±1.66	0.024
**7**	establishing intraoperative monitoring (ecg, non-invasive blood pressure monitoring, temperature, relaxometry, pulse oximetry/oxygen saturation)	**Doctors in Training**		**Consultant**		
		5.00 (2.00)	4.65 ±1.35	4.00 (3.00)	4.23 ±1.49	<0.001
		**Doctors in Training**		**Chief physician**		

		5.00 (2.00)	4.65 ±1.35	4.00 (2.00)	3.90 ±1.56	<0.001
		**Doctors in Training**		**Retired physician**		
		5.00 (2.00)	4.65 ±1.35	4.00 (2.00)	3.85 ±1.55	<0.001
		**Specialist**		**Chief physician**		
		5.00 (2.00)	4.41 ±1.45	4.00 (2.00)	3.90 ±1.56	<0.001
		**Specialist**		**Retired physician**		
		5.00 (2.00)	4.41 ±1.45	4.00 (2.00)	3.85 ±1.55	0.001
**8**	setting up an iv-drip for infusion	**Doctors in Training**		**Chief physician**		
		6.00 (1.00)	5.21 ±1.25	5.00 (2.00)	4.77 ±1.45	0.001
		**Doctors in Training**		**Retired physician**		
		6.00 (1.00)	5.21 ±1.25	5.00 (2.00)	4.83 ±1.36	0.011
		**Specialist**		**Chief physician**		
		6.00 (1.00)	5.13 ±1.27	5.00 (2.00)	4.77 ±1.45	0.003
		**Consultant**		**Chief physician**		
		6.00 (2.00)	5.06 ±1.35	5.00 (2.00)	4.77 ±1.45	0.025
**10**	establishing a peripheral iv catheter	**Doctors in Training**		**Chief physician**		
		6.00 (1.00)	5.36 ±1.08	5.00 (2.00)	4.78 ±1.40	<0.001
		**Doctors in Training**		**Retired physician**		
		6.00 (1.00)	5.36 ±1.08	5.00 (2.00)	4.66 ±1.44	<0.001
		**Specialist**		**Chief physician**		
		6.00 (1.00)	5.18 ±1.20	5.00 (2.00)	4.78 ±1.40	<0.001
		**Specialist**		**Retired physician**		
		6.00 (1.00)	5.18 ±1.20	5.00 (2.00)	4.66 ±1.44	<0.001

		Consultant		Chief physician		
		6.00 (2.00)	5.12 ±1.23	5.00 (2.00)	4.78 ±1.40	0.003
		Consultant		Retired physician		
		6.00 (2.00)	5.12 ±1.23	5.00 (2.00)	4.66 ±1.44	0.002
**11**	establishing a central iv catheter	**Doctors in Training**		**Specialist**		
		2.00 (2.00)	2.07 ±1.05	2.00 (2.00)	1.88 ±1.07	0.007
		**Doctors in Training**		**Consultant**		
		2.00 (2.00)	2.07 ±1.05	2.00 (1.00)	1.85 ±1.05	0.002
		**Doctors in Training**		**Chief physician**		
		2.00 (2.00)	2.07 ±1.05	1.00 (1.00)	1,73 ±1.05	<0.001
		**Doctors in Training**		**Retired physician**		
		2.00 (2.00)	2.07 ±1.05	1.00 (1.00)	1.87 ±1.20	0.0031
**12**	establishing an arterial catheter	**Doctors in Training**		**Specialist**		
		2.00 (2.00)	2.16 ±1.12	2.00 (2.00)	1.82 ±1.04	<0.001
		**Doctors in Training**		**Consultant**		
		2.00 (2.00)	2.16 ±1.12	2.00 (1.00)	1.82 ±1.04	<0.001
		**Doctors in Training**		**Chief physician**		
		2.00 (2.00)	2.16 ±1.12	1.00 (1.00)	1.70 ±1.10	<0.001
		**Doctors in Training**		**Retired physician**		
		2.00 (2.00)	2.16 ±1.12	1.00 (1.00)	1.74 ±1.22	<0.001
**13**	applying drugs intravenously, intramuscularly, subcutaneously	**Doctors in training**		**Specialist**		

		5.00 (2.00)	4.79 ±1.47	5.00 (2.00)	4.49 ±1.51	0.028
**14**	being capable of a sufficient preoxygenation	**Specialist**		**Chief physician**		
		4.00 (3.00)	3.77 ±1.62	3.00 (3.00)	3.42 ±1.65	0.037
**15**	being able to induce a general anaesthesia using hypnotics, opioids and muscle relaxants with adequate dosing	**Doctors in training**		**Chief physician**		
		2.00 (2.00)	2.03 ±1.13	1.00 (1.00)	1.83 ±1.21	0.018
**16**	being able to open the upper respiratory tract by using the Esmarch manoeuvre	**Doctors in training**		**Specialist**		
		5.00 (3.00)	4.33 ±1.70	5.00 (2.00)	4.67 ±1.56	0.047
		**Specialist**		**Chief physician**		
		5.00 (2.00)	4.67 ±1.56	5.00 (3.00)	4.31 ±1.64	0.021
**17**	being capable of ventilating a patient with a face mask (may be using a supraglottic airway tube)	**Specialist**		**Chief physician**		
		4.50 (3.00)	4.22 ±1.67	4.00 (4.00)	3.81 ±1.70	0.01
		**Specialist**		**Retired physician**		
		4.50 (3.00)	4.22 ±1.67	4.00 (3.00)	3.70 ±1.73	0.014
**18**	knowing how to correctly insert a laryngeal mask airway and checking for its correct positioning	**Specialist**		**Chief physician**		
		3.00 (2.00)	2.91 ±1.52	2.00 (3.00)	2.60 ±1.54	0.026
**19**	knowing how to correctly insert a laryngeal tube and checking for its correct positioning	**Specialist**		**Chief physician**		
		3.00 (3.00)	2.83 ±1.59	2.00 (3.00)	2.45 ±1.57	0.003
**22**	setting up an adequate mechanical ventilation according to the patient and the operation	**Doctors in training**		**Chief physician**		
		2.00 (2.00)	1.95 ±1.07	1.00 (1.00)	1.74 ±1.13	0.022
		**Specialist**		**Chief physician**		
		2.00 (2.00)	1.93 ±1.15	1.00 (1.00)	1.74 ±1.13	0.045
**23**	taking patient history focused on pain symptoms	**Doctors in training**		**Consultant**		
		4.00 (2.00)	4.07 ±1.43	4.00 (2.00)	3.70 ±1.48	0.004

		**Doctors in training**		**Chief physician**		
		4.00 (2.00)	4.07 ±1.43	3.00 (3.00)	3.56 ±1.46	<0.001
**24**	setting up a therapy plan according to the WHO analgesic ladder	**Doctors in training**		**Specialist**		
		4.00 (2.00)	3.93 ±1.47	4.00 (3.00)	3.60 ±1.51	0.026
		**Doctors in training**		**Consultant**		
		4.00 (2.00)	3.93 ±1.47	4.00 (3.00)	3.61 ±1.50	0.021
		**Doctors in training**		**Retired physician**		
		4.00 (2.00)	3.93 ±1.47	3.00 (2.00)	3.41 ±1.49	0.013
**29**	an interscalene approach to the brachial plexus	**Doctors in training**		**Retired physician**		
		1.00 (0.00)	1.10 ±0.34	1.00 (0.00)	1.27 ±0.71	0.026
		**Consultant**		**Retired physician**		
			1.12	1.00 (0.00)	1.27 ±0.71	0.007
**30**	a supraclavicular approach to the brachial plexus	**Doctors in training**		**Retired physician**		
		1.00 (0.00)	1.08 ±0.31	1.00 (0.00)	1.26 ±0.73	0.023
		**Consultant**		**Retired physician**		
		1.00 (0.00)	1.11 ±0.44	1.00 (0.00)	1.26 ±0.73	0.01
**31**	an axillary approach to the brachial plexus	**Doctors in training**		**Retired physician**		
		1.00 (0.00)	1.12 ±0.42	1.00 (0.00)	1.43 ±0.97	0.001
		**Consultant**		**Retired physician**		
		1.00 (0.00)	1.15 ±0.52	1.00 (0.00)	1.43 ±0.97	<0.001
**32**	blocking the femoral nerve	**Doctors in training**		**Retired physician**		

		1.00 (0.00)	1.14 ±0.43	1.00 (0.00)	1.40 ±0.88	0.002
		**Consultant**		**Retired physician**		
		1.00 (0.00)	1.17 ±0.55	1.00 (0.00)	1.40 ±0.88	0.001
**35**	performing a clinically focused physical exam	**Doctors in training**		**Consultant**		
		6.00 (2.00)	5.02 ±1.24	5.00 (2.00)	4.72 ±1.37	0.016
		**Doctors in training**		**Retired physician**		
		6.00 (2.00)	5.02 ±1.24	5.00 (3.00)	4.34 ±1.48	<0.001
		**Specialist**		**Retired physician**		
		5.00 (2.00)	4.74 ±1.44	5.00 (3.00)	4.34 ±1.48	0.021
**36**	transferring information regarding a patient among healthcare professionals utilizing a defined technique (for example SBAR)	**Doctors in Training**		**Specialist**		
		5.00 (2.00)	4.58 ±1.44	4.00 (3.00)	4.19 ±1.60	0,008
		**Doctors in Training**		**Consultant**		
		5.00 (2.00)	4.58 ±1.44	4.00 (3.00)	4.16 ±1.59	0,002
		**Doctors in Training**		**Chief physician**		
		5.00 (2.00)	4.58 ±1.44	4.00 (3.00)	3.71 ±1.71	<0.001
		**Doctors in Training**		**Retired physician**		
		5.00 (2.00)	4.58 ±1.44	4.00 (3.00)	3.54 ±1.61	<0.001
		**Specialist**		**Chief physician**		
		4.00 (3.00)	4.19 ±1.60	4.00 (3.00)	3.71 ±1.71	0.002
		**Specialist**		**Retired physician**		
		4.00 (3.00)	4.19 ±1.60	4.00 (3.00)	3.54 ±1.61	0.001
		**Consultant**		**Chief physician**		

		4.00 (3.00)	4.16 ±1.59	4.00 (3.00)	3.71 ±1.71	0.004
		**Consultant**		**Retired physician**		
		4.00 (3.00)	4.16 ±1.59	4.00 (3.00)	3.54 ±1.61	0.001
**37**	managing an analgosedation for an intervention	**Doctors in training**		**Consultant**		
		2.00 (2.00)	2.34 ±1.17	2.00 (2.00)	2.11 ±1.20	0.008
		**Doctors in training**		**Chief physician**		
		2.00 (2.00)	2.34 ±1.17	2.00 (2.00)	2.07 ±1.25	0.01
**39**	demonstrating ultrasound examination utilizing the eFAST principle	**Doctors in training**		**Specialist**		
		3.00 (2.00)	2.98 ±1.62	2.00 (2.00)	2.42 ±1.45	<0.001
		**Doctors in training**		**Consultant**		
		3.00 (2.00)	2.98 ±1.62	2.00 (2.00)	2.48 ±1.47	<0.001
		**Doctors in training**		**Chief physician**		
		3.00 (2.00)	2.98 ±1.62	2.00 (2.00)	2.42 ±1.50	<0.001
		**Doctors in training**		**Retired physician**		
		3.00 (2.00)	2.98 ±1.62	2.00 (2.00)	2.27 ±1.44	<0.001
**40**	inserting a gastric tube	**Specialist**		**Retired physician**		
		3.00 (3.00)	3.42 ±1.62	4.00 (4.00)	3.87 ±1.65	0.059
		**Chief physician**		**Retired physician**		
		3.00 (3.00)	3.28 ±1.69	4.00 (4.00)	3.87 ±1.65	0.009
**46**	obtaining blood samples for microbiological examination	**Doctors in training**		**Specialist**		
		5.00 (2.00)	4.91 ±1.37	5.00 (3.00)	4.44 ±1.59	0.001

		**Doctors in training**		**Consultant**		
		5.00 (2.00)	4.91 ±1.37	5.00 (3.00)	4.54 ±1.55	0.014
		**Doctors in training**		**Chief physician**		
		5.00 (2.00)	4.91 ±1.37	5.00 (3.00)	4.24 ±1.64	<0.001
		**Doctors in training**		**Retired physician**		
		5.00 (2.00)	4.91 ±1.37	5.00 (3.00)	4.30 ±1.61	0.004
**47**	performing a blood transfusion according to current guidelines	**Doctors in training**		**Specialist**		
		5.00 (2.00)	4.75 ±1.58	5.00 (3.00)	4.30 ±1.75	0.005
		**Doctors in training**		**Consultant**		
		5.00 (2.00)	4.75 ±1.58	5.00 (3.00)	4.27 ±1.79	0.002
		**Doctors in training**		**Chief physician**		
		5.00 (2.00)	4.75 ±1.58	5.00 (3.00)	4.13 ±1.83	0.001
		**Doctors in training**		**Retired physician**		
		5.00 (2.00)	4.75 ±1.58	4.00 (4.00)	3.97 ±1.86	0.001
**49**	inspecting a corpse externally	**Doctors in training**		**Consultant**		
		5.00 (3.00)	4.36 ±1.68	4.00 (4.00)	3.88 ±1.88	0.007
		**Doctors in training**		**Chief physician**		
		5.00 (3.00)	4.36 ±1.68	4.00 (4.00)	3.80 ±1.95	0.011
**51**	communication adequately with patients and/or relatives in crisis situations	**Doctors in training**		**Chief physician**		
		4.00 (3.00)	3.98 ±1.72	3.00 (3.00)	3.47 ±1.80	0.009

**52**	calculating the Glasgow Coma Scale	**Doctors in training**		**Retired physician**		
		6.00 (2.00)	4.95 ±1.32	4.00 (3.00)4	4.28 ±1.63	0.001
		**Specialist**		**Retired physician**		
		6.00 (2.00)	4.87 ±1.43	4.00 (3.00)	4.28 ±1.63	0.001
		**Consultant**		**Retired physician**		
		6.00 (2.00)	4.94 ±1.37	4.00 (3.00)	4.28 ±1.63	<0.001
**53**	evaluating a patient using the ABCDE system	**Doctors in training**		**Chief physician**		
		6.00 (2.00)	4.96 ±1.35	5.00 (3.00)	4.47 ±1.61	0.007
		**Doctors in training**		**Retired physician**		
		6.00 (2.00)	4.96 ±1.35	4.00 (3.00)	4.18 ±1.59	<0.001
		**Specialist**		**Retired physician**		
		5.00 (2.00)	4.79 ±1.47	4.00 (3.00)	4.18 ±1.59	<0.001
		**Consultant**		**Retired physician**		
		5.00 (2.00)	4.76 ±1.48	4.00 (3.00)	4.18 ±1.59	0.001
**54**	performing a rapid sequence induction and intubation	**Specialist**		**Chief physician**		
		1.00 (1.00)	1.87 ±1.18	1.00 (1.00)	1.72 ±1.25	0.049
**55**	establishing an intraosseous needle	**Specialist**		**Chief physician**		
		2.00 (3.00)	2.76 ±1.64	2.00 (2.00)	2.40 ±1.51	0.029
**58**	immobilizing a patient using a vacuum mattress or spineboard	**Doctors in Training**		**Specialist**		
		3.00 (3.00)	3.50 ±1.64	4.00 (3.00)	3.96 ±1.68	0.001
		**Doctors in Training**		**Consultant**		
		3.00 (3.00)	3.50 ±1.64	4.00 (3.00)	3.94 ±1.71	0.002
		**Doctors in Training**		**Chief physician**		

		3.00 (3.00)	3.50 ±1.64	4.00 (3.00)	4.03 ±1.72	0.002
		**Doctors in Training**		**Retired physician**		
		3.00 (3.00)	3.50 ±1.64	5.00 (3.00)	4.42 ±1.67	<0.001
		**Specialist**		**Retired physician**		
		4.00 (3.00)	3.96 ±1.68	5.00 (3.00)	4.42 ±1.67	0.049
		**Consultant**		**Retired physician**		
		4.00 (3.00	3.94 ±1.714	5.00 (3.00)	4.42 ±1.67	0.032
**60**	placing a tourniquet	**Doctors in training**		**Retired physician**		
		4.00 (3.00)	3.66 ±1.74	5.00 (3.00)	4.38 ±1.71	0.001
		**Consultant**		**Retired physician**		
		4.00 (4.00)	3.87 ±1.74	5.00 (3.00)	4.38 ±1.71	0.031
		**Chief physician**		**Retired physician**		
		4.00 (3.00)	3.79 ±1.68	5.00 (3.00)	4.38 ±1.71	0.02
**62**	utilizing the basic life support algorithms according to current guidelines and performing effective chest compressions	**Doctors in training**		**Retired physician**		
		6.00 (0.00)	5.54 ±1.03	6.00 (1.00)	5.21 ±1.26	0.031
**63**	correctly analysing the different rhythms in cardiac arrest	**Doctors in training**		**Consultant**		
		6.00 (2.00)	4.87 ±1.45	5.00 (3.00)	4.54 ±1.53	0.009
		**Doctors in training**		**Chief physician**		
		6.00 (2.00)	4.87 ±1.45	5.00 (3.00)	4.48 ±1.55	0.02
		**Doctors in training**		**Retired physician**		
		6.00 (2.00)	4.87 ±1.45	5.00 (3.00)	4.36 ±1.61	0.018
**64**	correctly perform defibrillation/cardioversion	**Doctors in training**		**Chief physician**		

		6.00 (2.00)	4.86 ±1.49	5.00 (3.00)	4.44 ±1.69	0.021
		**Doctors in training**		**Retired physician**		
		6.00 (2.00)	4.86 ±1.49	5.00 (3.00)	4.37 ±1.67	0.045
**65**	correctly administer drugs	**Doctors in training**		**Chief physician**		
		5.00 (2.00)	4.76 ±1.54	5.00 (3.00)	4.38 ±1.64	0.04

2. Significant differences in the evaluation depending on the workplace

The following table shows an overview of significant differences in the evaluation of individual learning objectives depending on the workplace of the surveyed physicians.

**item**	**competence**	**level of care 1**		**level of care 2**		**p**
		**median (IQR)**	**mean ±SD**	**median (IQR)**	**mean ±SD**	
**Comparison of the different level of care**						
At the end of undergraduate training, the student, as an active member of the professional team, can safely carry out clinical-practical skills adequately and independently under supervision, in a manner that is respectful of the patient. The student can…						
**1**	taking patient history relevant to anaesthesia	**Regional level of care**		**University hospital**		
		3.00 (2.00)	3.19 ±1.38	3.00 (2.00)	3.53 ±1.41	0.002
		**Overregional**		**University hospital**		
		3.00 (2.00)	3.15 ±1.32	3.00 (2.00)	3.53 ±1.41	0.002
		**Overregional**		**Outpatient practice**		
		3.00 (2.00)	3.15 ±1.32	3.00 (2.00)	3.53 ±1.42	0.047
		**Maximum level of care**		**University hospital**		

		3.00 (2.00)	3.15 ±1.41	3.00 (2.00)	3.53 ±1.41	0.002
		**Maximum level of care**		**Outpatient practice**		
		3.00 (2.00)	3.15 ±1.41	3.00 (2.00)	3.53 ±1.42	0.034
**2**	performing an anaesthesia focused physical examination (auscultation of heart/lung, status of teeth, predictors of a difficult airway, …)	**Regional level of care**		**University hospital**		
		4.00 (2.75)	3.63 ±1.43	4.00 (2.00)	3.93 ±1.35	0.022
**3**	performing a 12-channel-ecg and interpretation of the result	**Outpatient practice**		**University hospital**		
		4.00 (3.00)	4.29 ±1.42	6.00 (2.00)	5.03 ±1.25	<0.001
**4**	conducting an informed consent discussion with an ASAI/ASAII patient undergoing a low to medium risk operation and documenting it in a legally correct manner	**Special supply**		**University hospital**		
		2.00 (2.00)	2.14 ±1.29	2.00 (1.00)	2.60 ±1.32	0.043
		**Special supply**		**Outpatient practice**		
		2.00 (2.00)	2.14 ±1.29	3.00 (2.00)	2.83 ±1.46	0.002
		**Maximum level of care**		**Outpatient practice**		
		2.00 (2.00)	2.39 ±1.30	3.00 (2.00)	2.83 ±1.46	0.009
**5**	performing a quick check of the anaesthesia working place according to the recommendations of DGAI	**Regional level of care**		**University hospital**		
		1.00 (1.00)	1.89 ±1.29	2.00 (2.00)	2.11 ±1.35	0.013
		**Overregional**		**University hospital**		
		1.00 (1.00)1	1.84 ±1.27	2.00 (2.00)	2.11 ±1.35	0.002
		**Overregional**		**Outpatient practice**		
		1.00 (1.00)	1.84 ±1.27	2.00 (2.00)	2.20 ±1.46	0.002
**7**	establishing intraoperative monitoring (ecg, non-invasive blood pressure	**Overregional**		**University hospital**		

	monitoring, temperature, relaxometry, pulse oximetry/oxygen saturation)	4.00 (3.00)	4.12 ±1.55	5.00 (2.00)	4.53 ±1.38	0.002
**10**	establishing a peripheral iv catheter	**Overregional**		**University hospital**		
		6.00 (2.00)	4.98 ±1.28	6.00 (1.00)	5.27 ±1.13	0.009
**12**	establishing an arterial catheter	**Special supply**		**University hospital**		
		1.00 (1.00)	1.56 ±0.98	2.00 (2.00)	1.97 ±1.10	0.013
		**Outpatient practice**		**University hospital**		
		1.00 (1.00)	1.65 ±0.94	2.00 (2.00)	1.97 ±1.10	0.007
**15**	being able to induce a general anaesthesia using hypnotics, opioids and muscle relaxants with adequate dosing	**Regional level of care**		**University hospital**		
		2.00 (3.00)	1.86 ±1.05	2.00 (2.00)	2.13 ±1.22	0.026
		**Overregional**		**University hospital**		
		1.00 (1.00)	1.87 ±1.16	2.00 (2.00)	2.13 ±1.22	0.006
**16**	being able to open the upper respiratory tract by using the Esmarch manoeuvre	**Overregional**		**University hospital**		
		5.00 (3.00)	4.29 ±1.66	5.00 (2.00)	4.66 ±1.53	0.033
		**Overregional**		**Outpatient practice**		
		5.00 (3.00)	4.29 ±1.66	6.00 (2.00)	4.86	0.001
**17**	being capable of ventilating a patient with a face mask (may be using a supraglottic airway tube)	**Overregional**		**University hospital**		
		4.00 (4.00)	3.82 ±1.75	5.00 (3.00)	4.29 ±1.68	0.001
		**Overregional**		**Outpatient practice**		
		4.00 (4.00)	3.82 ±1.75	4.00 (3.00)	4.86 ±1.55	0.048
**18**		**Overregional**		**University hospital**		

	knowing how to correctly insert a laryngeal mask airway and checking for its correct positioning	2.00 (3.00)	2.61 ±1.48	3.00 (2.00)	2.90 ±1.46	0.043
		**Overregional**		**Outpatient practice**		
		2.00 (3.00)	2.61 ±1.48	3.00 (2.00)	3.02 ±1.49	0.036
**19**	knowing how to correctly insert a laryngeal tube and checking for its correct positioning	**Regional level of care**		**Overregional**		
		3.00 (3.00)	2.83 ±1.60	2.00 (2.00)	2.38 ±1.47	<0.001
		**Overregional**		**Outpatient practice**		
		2.00 (2.00)	2.38 ±1.47	3.00 (2.00)	3.11 ±1.62	<0.001
		**Maximum level of care**		**Outpatient practice**		
		2.00 (3.00)	2.62 ±1.56	3.00 (2.00)	3.11 ±1.62	0.013
		**University hospital**		**Outpatient practice**		
		2.00 (3.00)	2.66 ±1.53	3.00 (2.00)	3.11 ±1.62	0.033
**20**	intubating a patient and checking for the correct endotracheal positioning	**Overregional**		**Outpatient practice**		
		2.00 (2.00)	1.99 ±1.21	2.00 (2.00)	2.39 ±1.34	0.003
**21**	performing the initial steps of an emergency algorithm when encountering an unexpected difficult airway	**Overregional**		**University hospital**		
		2.00 (2.00)	2.27 ±1.49	2.00 (2.00)	2.56 ±1.49	0.01
		**Overregional**		**Outpatient practice**		
		2.00 (2.00)	2.27 ±1.49	2.00 (3.00)	2.74 ±1.62	0.003
**22**	setting up an adequate mechanical ventilation according to the patient and the operation	**Regional level of care**		**University hospital**		
		1.00 (1.00)	1.78 ±1.04	2.00 (2.00)	2.06 ±1.18	0.002
		**Overregional**		**University hospital**		

		1.00 (1.00)	1.79 ±1.11	2.00 (2.00)	2.06 ±1.18	0.001
**24**	setting up a therapy plan according to the WHO analgesic ladder	**University hospital**		**Outpatient practice**		
		4.00 (3.00)	3.88 ±1.54	3.00 (2.00)	3.36 ±1.32	0.002
**31**	an axillary approach to the brachial plexus	**Regional level of care**		**Outpatient practice**		
		1.00 (0.00)	1.14 ±0.47	1.00 (0.00)	1.30 ±0.75	0.025
**35**	performing a clinically focused physical exam	**University hospital**		**Outpatient practice**		
		5.00 (2.00)	4.88 ±1.34	5.00 (3.00)	4.36 ±1.64	0.016

**36**	transferring information regarding a patient among healthcare professionals utilizing a defined technique (for example SBAR)	**Overregional**		**University hospital**		
		4.00 (3.00)	3.98 ±1.67	5.00 (3.00)	4.55 ±1.48	<0.001
		**Maximum level of care**		**University hospital**		
		4.00 (3.00)	4.17 ±1.54	5.00 (3.00)	4.55 ±1.48	0.03
		**Outpatient practice**		**University hospital**		
		4.00 (3.00)	3.65 ±1.65	5.00 (3.00)	4.55 ±1.48	<0.001
		**Other**		**University hospital**		
		4.00 (3.00)	3.85 ±1.66	5.00 (3.00)	4.55 ±1.48	0.049
		**Outpatient practice**		**Maximum level of care**		
		4.00 (3.00)	3.65 ±1.65	4.00 (3.00)	4.17 ±1.54	0.037
**39**	demonstrating ultrasound examination utilizing the eFAST principle	**Regional level of care**		**Outpatient practice**		
		2.00 (2.00)	2.47 ±1.47	2.00 (1.00)	1.97 ±1.25	0.001

		**Regional level of care**		**University hospital**		
		2.00 (2.00)	2.47 ±1.47	3.00 (3.00)	2.77 ±1.49	0.032
		**Overregional**		**University hospital**		
		2.00 (2.00)	2.47 ±1.52	3.00 (3.00)	2.77 ±1.49	0.025
		**Overregional**		**Outpatient practice**		
		2.00 (2.00)	2.47 ±1.52	2.00 (1.00)	1.97 ±1.25	0.004
		**Maximum level of care**		**Outpatient practice**		
		2.00 (3.00)	2.63 ±1.57	2.00 (1.00)	1.97 ±1.25	<0.001
		**University hospital**		**Outpatient practice**		
		3.00 (3.00)	2.77 ±1.49	2.00 (1.00)	1.97 ±1.25	<0.001
**46**	obtaining blood samples for microbiological examination	**Regional level of care**		**Outpatient practice**		
		5.00 (2.00)	4.60 ±1.52	4.00 (3.00)	4.13 ±1.67	0.035
		**Maximum level of care**		**Outpatient practice**		
		5.00 (3.00)	4.65 ±1.51	4.00 (3.00)	4.13 ±1.67	0.021
		**University hospital**		**Outpatient practice**		
		5.00 (2.00)	4.59 ±1.57	4.00 (3.00)	4.13 ±1.67	0.04
**47**	performing a blood transfusion according to current guidelines	**University hospital**		**Outpatient practice**		
		5.00 (3.00)	4.52 ±1.73	4.00 (4.00)	3.96 ±1.82	0.013
**52**	calculating the Glasgow Coma Scale	**Maximum level of care**		**Outpatient practice**		
		6.00 (2.00)	4.92 ±1.47	5.00 (3.00)	4.48 ±1.60	0.022

		**University hospital**		**Outpatient practice**		
		6.00 (2.00)	5.07 ±1.29	5.00 (3.00)	4.48 ±1.60	0.001
**53**	evaluating a patient using the ABCDE system	**Overregional**		**University hospital**		
		5.00 (3.00)	4.63 ±1.52	6.00 (2.00)	5.00 ±1.31	0.017
		**Maximum level of care**		**Outpatient practice**		
		6.00 (2.00)	4.82 ±1.50	5.00 (3.00)	4.38 ±1.60	0.038
		**University hospital**		**Outpatient practice**		
		6.00 (2.00)	5.00 ±1.31	5.00 (3.00)	4.38 ±1.60	<0.001
**54**	performing a rapid sequence induction and intubation	**Overregional**		**University hospital**		
		1.00 (1.00)	1.63 ±1.01	1.50 (1.00)	1.87 ±1.11	0.011
		**Overregional**		**Outpatient practice**		
		1.00 (1.00)	1.63 ±1.01	2.00 (2.00)	2.03 ±1.33	0.017
**58**	immobilizing a patient using a vacuum mattress or spineboard	**Overregional**		**Outpatient practice**		
		4.00 (4.00)	3.80 ±1.73	5.00 (3.00)	4.31 ±1.33	0.021
		**Outpatient practice**		**University hospital**		
		5.00 (3.00)	4.31 ±1.69	4.00 (3.00)	3.73 ±1.62	0.003
**63**	correctly analysing the different rhythms in cardiac arrest	**Regional level of care**		**University hospital**		
		5.00 (2.00)	4.57 ±1.50	6.00 (2.00)	4.89 ±1.44	0.015
		**Outpatient practice**		**University hospital**		
		5.00 (3.00)	4.46 ±1.51	6.00 (2.00)	4.89 ±1.44	0.021

**64**	correctly perform defibrillation/cardioversion	**Overregional**		**University hospital**		
		5.00 (3.00)	4.46 ±1.63	6.00 (2.00)	4.87 ±1.47	0.003
**65**	correctly administer drugs	**Overregional**		**University hospital**		
		5.00 (3.00)	4.34 ±1.62	6.00 (2.00)	4.87 ±1.50	<0.001

3. Significant differences in the evaluation depending additional certifications

The following table shows an overview of significant differences in the assessment of individual learning objectives depending on whether the physicians surveyed had an additional qualification in a specific area (e.g. emergency medicine, intensive care medicine).

a) Physicians with additional certification in emergency medicine

**item**	**competence**	**Certification yes**		**Certification no**		**p**
		**median (IQR)**	**mean ±SD**	**median (IQR)**	**mean ±SD**	
**Comparison of the different level of care**						
At the end of undergraduate training, the student, as an active member of the professional team, can safely carry out clinical-practical skills adequately and independently under supervision, in a manner that is respectful of the patient. The student can…						
**9**	preparing drugs for intravenous application	6.00 (2.00)	4.95 ±1.37	6.00 (2.00)	4.77 ±1.50	0.036
**11**	establishing a central iv catheter	2.00 (1.00)	1.85 ±1.08	2.00 (2.00)	1.96 ±1.04	0.003
**12**	establishing an arterial catheter	1.00 (1.00)	1.82 ±1.08	2.00 (2.00)	1.94 ±1.06	0.003
**21**	performing the initial steps of an emergency algorithm when encountering an unexpected difficult airway	2.00 (2.00)	2.40 ±1.51	2.00 (2.00)	2.51 ±1.48	0.026
**23**	taking patient history focused on pain symptoms	4.00 (3.00)	3.71 ±1.49	4.00 (2.00)	3.91 ±1.48	0.005

b) Physicians with additional certification in intensive care medicine

item	competence	Certification yes		Certification no		p
		median (IQR)	mean ±SD	median (IQR)	mean ±SD	
**Comparison of the different level of care**						
At the end of undergraduate training, the student, as an active member of the professional team, can safely carry out clinical-practical skills adequately and independently under supervision, in a manner that is respectful of the patient. The student can…						
**7**	establishing intraoperative monitoring (ecg, non-invasive blood pressure monitoring, temperature, relaxometry, pulse oximetry/oxygen saturation)	4.00 (2.00)	4.11 ±1.52	5,00 (3.00)	4,44 ±1.44	<0.001
**8**	setting up an iv-drip for infusion	6.00 (2.00)	4.94 ±1.40	6,00 (1.00)	5.15 ±1.26	<0.001
**10**	establishing a peripheral iv catheter	6.00 (2.00)	5.03 ±1.27	6.00 (1.00)	5.16 ±1.23	0.003
**11**	establishing a central iv catheter	1.00 (1.00)	1.81 ±1.05	2.00 (2.00)	1.94 ±1.08	0.001
**12**	establishing an arterial catheter	1.00 (1.00)	1.76 ±1.06	2.00 (2.00)	1.93 ±1.08	<0.001
**14**	being capable of a sufficient preoxygenation	3.00 (3.00)	3.46 ±1.67	4.00 (3.00)	3.73 ±1.60	<0.001
**15**	being able to induce a general anaesthesia using hypnotics, opioids and muscle relaxants with adequate dosing	2.00 (1.00)	1.91 ±1.16	2.00 (2.00)	2.01 ±1.16	0.01
**18**	knowing how to correctly insert a laryngeal mask airway and checking for its correct positioning	2.00 (3.00)	2.74 ±1.54	3.00 (2.00)	2.85 ±1.51	0.046
**19**	knowing how to correctly insert a laryngeal tube and checking for its correct positioning	2.00 (3.00)	2.57 ±1.55	2.00 (3.00)	2.78 ±1.59	0.002
**21**	performing the initial steps of an emergency algorithm when encountering an unexpected difficult airway	2.00 (2.00)	2.33 ±1.50	2.00 (2.00)	2.51 ±1.49	0.001
**22**	setting up an adequate mechanical ventilation according to the patient and the operation	1.00 (1.00)	1.82 ±1.12	2.00 (2.00)	1.95 ±1.12	0.001

**23**	taking patient history focused on pain symptoms	4.00 (2.00)	3.68 ±1.49	4.00 (2.00)	3.84 ±1.49	0.016
**36**	transferring information regarding a patient among healthcare professionals utilizing a defined technique (for example SBAR)	4.00 (3.00)	4.01 ±1.63	4.00 (3.00)	4.24 ±1.59	0.003
**37**	managing an analgosedation for an intervention	2.00 (2.00)	2.08 ±1.21	2.00 (2.00)	2.22 ±1.20	0.001
**49**	inspecting a corpse externally	4.00 (4.00)	3.83 ±1.92	4.00 (3.00)	4.08 ±1.80	0.006
**51**	communication adequately with patients and/or relatives in crisis situations	4.00 (3.00)	3.60 ±1.75	4.00 (4.00)	3.83 ±1.71	0.004
**54**	performing a rapid sequence induction and intubation	1.00 (1.00)	1.71 ±1.12	1.00 (1.00)	1.86 ±1.14	<0.001
**55**	establishing an intraosseous needle	2.00 (3.00)	2.54 ±1.61	2.00 (3.00)	2.70 ±1.58	0.006

c) Physicians with additional certification in palliative care

**item**	**competence**	**Certification yes**		**Certification no**		**p**
		**median (IQR)**	**mean ±SD**	**median (IQR)**	**mean ±SD**	
**Comparison of the different level of care**						
At the end of undergraduate training, the student, as an active member of the professional team, can safely carry out clinical-practical skills adequately and independently under supervision, in a manner that is respectful of the patient. The student can…						
**29**	an interscalene approach to the brachial plexus	1.00 (0.00)	1.05 ±0.24	1.00 (0.00)	1.15 ±0.53	0.003
**30**	a supraclavicular approach to the brachial plexus	1.00 (0.00)	1.05 ±0.24	1.00 (0.00)	1.14 ±0.50	0.008
**31**	an axillary approach to the brachial plexus	1.00 (0.00)	1.13 ±0.49	1.00 (0.00)	1.20 ±0.62	0.045
**48**	calling a patient’s death	6.00 (3.00)	4.71 ±1.68	5.00 (3.00)	4.47 ±1.72	0.026

d) Physicians with additional certification in pain medicine

item	competence	Certification yes		Certification no		p
		median (IQR)	mean ±SD	median (IQR)	mean ±SD	
**Comparison of the different level of care**						
At the end of undergraduate training, the student, as an active member of the professional team, can safely carry out clinical-practical skills adequately and independently under supervision, in a manner that is respectful of the patient. The student can…						
**7**	establishing intraoperative monitoring (ecg, non-invasive blood pressure monitoring, temperature, relaxometry, pulse oximetry/oxygen saturation)	4.00 (2.00)	4.08 ±1.50	5.00 (3.00)	4.32 ±1.48	0.01
**23**	taking patient history focused on pain symptoms	4.00 (2.50)	3.59 ±1.42	4.00 (2.00)	3.79 ±1.50	0.025
**35**	performing a clinically focused physical exam	4.00 (2.00)	4.45 ±1.48	5.00 (2.00)	4.76 ±1.40	0.001
**36**	transferring information regarding a patient among healthcare professionals utilizing a defined technique (for example SBAR)	4.00 (2.00)	3.89 ±1.60	4.00 (3.00)	4.17 ±1.61	0.004
**39**	demonstrating ultrasound examination utilizing the eFAST principle	2.00 (2.00)	2.33 ±1.40	2.00 (3.00)	2.54 ±1.52	0.042
**46**	obtaining blood samples for microbiological examination	4.00 (3.00)	4.23 ±1.64	5.00 (3.00)	4.55 ±1.55	0.001
**52**	calculating the Glasgow Coma Scale	5.00 (3.00)	4.60 ±1.57	6.00 (2.00)	4.89 ±1.40	0.006
**53**	evaluating a patient using the ABCDE system	5.00 (3.00)	4.44 ±1.63	5.00 (2.00)	4.78 ±1.46	0.002
**55**	establishing an intraosseous needle	2.00 (2.00)	2.46 ±1.62	2.00 (3.00)	2.65 ±1.59	0.019
**62**	utilizing the basic life support algorithms according to current guidelines and performing effective chest compressions	6.00 (1.00)	5.21 ±1.36	6.00 (1.00)	5.42 ±1.15	0.011
**63**	correctly analysing the different rhythms in cardiac arrest	5.00 (3.00)	4.43 ±1.51	5.00 (2.00)	4.64 ±1.52	0.016
**64**	correctly perform defibrillation/cardioversion	5.00 (3.00)	4.42 ±1.62	5.00 (2.00)	4.65 ±1.56	0.021
**65**	correctly administer drugs	5.00 (3.00)	4.35 ±1.63	5.00 (3.00)	4.59 ±1.58	0.019
